# The impact of remuneration, extrinsic and intrinsic incentives on interprofessional primary care teams: results from a rapid scoping review

**DOI:** 10.1186/s12875-024-02653-5

**Published:** 2025-02-04

**Authors:** Monica Aggarwal, Brian Hutchison, Kristina M. Kokorelias, Selin Bilgic, Richard H. Glazier

**Affiliations:** 1 Dalla Lana School of Public Health, University of Toronto, 155 College St, Room 500, Toronto, ON M5T 3M7 Canada; 2 Department of Family and Community Medicine, University of Toronto, Toronto, Canada; 3 Departments of Family Medicine and Health Research Methods, Evidence and Impact, McMaster, Hamilton, Canada; 4 Department of Medicine, University Health Network, Toronto, ON Canada; 5 Department of Medicine, Sinai Health System, Toronto, ON Canada; 6 Department of Occupational Science & Occupational Therapy and Rehabilitation Sciences Institute, Temerty Faculty of Medicine, University of Toronto, Toronto, ON Canada; 7 Institute for Clinical Evaluative Sciences, Toronto, ON Canada; 8 Michael’s Hospital, Toronto, ON Canada

**Keywords:** Teams, Primary care, Remuneration, Motivation, Interprofessional, Funding models, Incentives

## Abstract

**Background:**

High-performing primary care relies on effective interprofessional teams and provider payment arrangements. This study aims to examine the impact of provider remuneration mechanisms and intrinsic and extrinsic incentives in team-based primary care.

**Methods:**

This rapid scoping review assessed various provider payment models and incentives in team-based primary care. Statistical tests were not applicable in this review.

**Results:**

Fee-for-service models hindered team collaboration, while salaried and quality-based compensation models enhanced collaboration. Extrinsic incentives, such as pay-for-performance programs for physicians, showed mixed impacts on outcomes. Strong organizational cultures and leadership, resources, team meetings, training, clear protocols, and professional development opportunities facilitated teamwork. Intrinsic incentives like autonomy, mastery, and social purpose improved team performance and satisfaction.

**Conclusions:**

This study underscores the importance of a holistic approach to designing interprofessional primary care teams. It highlights the need for implementing non-fee-for-service provider payment models and team-based pay-for-performance incentives. Investments in teams should include health human resources and leadership, training, guidelines, and professional development opportunities. Implementing a performance measurement framework for teams and regular public reporting can foster mastery. Continuous research and evaluation are crucial to optimizing teamwork and healthcare delivery in primary care settings.

**Supplementary Information:**

The online version contains supplementary material available at 10.1186/s12875-024-02653-5.

## Background

Globally, primary care (PC) systems are in crisis, with reports of shortages in the PC workforce and challenges with patient access [1–3]. The spread of interprofessional PC teams is recognized as one solution to the global crisis [1]. In Canada, several jurisdictions have introduced team-based models, which vary in their organizational structure, remuneration schemes, provider composition, governance mechanisms, enrolment of patients, and target population [4]. Interprofessional PC teams have a positive impact on patient health outcomes [5], patient satisfaction [6], reducing the number of hospitalizations, and emergency room visits - fostering cost-saving [7].

Interprofessional teams and funding and payment provider arrangements aligned with health system goals are key attributes of high-performing primary care (PC) systems [4, 8]. PC teams are a group of professionals from two or more disciplines that work interdependently to deliver patient care [9, 10]. Globally, several jurisdictions have embarked on the implementation and spread of PC teams [1]. Investments in team-based care have been made in Canada [11, 12], Brazil [13], Norway [14, 15], the United States [16, 17], Australia [18, 19], and New Zealand [20]. The organizational models vary in terms of team composition, populations served, funding and remuneration payment mechanisms and requirements for attachment [1, 21].

The evidence of impact on PC teams in Canada is also mixed and limited [22, 23], particularly in mature models such as in Alberta, Ontario, and Quebec [23, 24]. In Alberta, studies show that Primary Care Networks (PCNs) reduce emergency department visits while others demonstrate declines in care coordination and comprehensiveness [24–28]. In Quebec’s Family Medicine Groups (FMGs), the results are mixed with respect to impact on service use, equity, and access [29–36] and show no changes in chronic condition screening [37] or adherence to medication guidelines [38]. In Ontario, Family Health Teams (FHTs) have been shown to improve same-day access to care but show mixed results on the impact on emergency department visits and no impact on after-hours care or hospital admissions [39, 40].

Physician payment models refer to the various structures through which healthcare providers are compensated for their services [41]. A variety of physician payment models (fee-for-service (FFS), capitation, salary, blended payment) are used in PC teams [23]. A systematic review found salaried payment is associated with the lower use of tests, number of procedures and patients per doctor, and referrals but longer consultations and more preventive care compared with FFS [42]. However, the impact of various remuneration models on team collaboration or effectiveness is unknown. Extrinsic incentives provided by organizations and intrinsic incentives driven from within an individual have been demonstrated to improve team performance [23, 43–46]. However, their impact has not been studied in PC [23].

Remuneration models have been thought to impact team functioning [47] and the delivery of care within interprofessional PC teams [48]. For example, under an FFS model, physicians are reimbursed based on the volume of services rendered. This can impact team dynamics by prioritizing income over team collaboration [49]. Research has also shown that extrinsic and intrinsic motivators can increase patient satisfaction, improve clinical outcomes, and lead to more efficient healthcare delivery [50], including within interprofessional teams [51, 52]. Extrinsic incentives (pay raises, bonuses, professional development) can enhance team performance, quality of care and provider well-being [23, 53], and help with the retention of skilled professionals, ensuring continuity and consistency in patient care [54]. Intrinsic incentives (autonomy, advancement opportunity, personal satisfaction) can increase job satisfaction, engagement in work, and the sense of personal accomplishment [55–57].

To our knowledge, a comprehensive review has yet to be done to examine the impact of remuneration models, extrinsic and intrinsic incentives on patient, provider, team, and system outcomes in PC. Incentives refer to rewards or inducements, often financial or non-financial, offered to individuals or teams to motivate specific behaviors, actions, or outcomes. "Team outcomes" refers to the specific results or effects that are directly attributable to the functioning, collaboration, and performance of interprofessional teams within primary care settings.

In the evolving landscape of healthcare delivery, where team-based care is heralded as crucial to alleviating the PC crisis, this review can inform the design of team-based models, policy-making, and healthcare management [23]. This review aims to examine the impact of provider remuneration models and extrinsic and intrinsic team-based incentives on various outcomes in PC [23]. In the context of PC, the central research objectives of this scoping review are to: (a) examine the impact of provider (physician or nurse practitioner-led) remuneration models on outcomes; (b) identify extrinsic team-based incentives and their impact on outcomes; (c) identify intrinsic team-based incentives and their impact on outcomes.

## Methods

### Design

We conducted a rapid scoping review [58, 62] using the Arksey and O’Malley framework [59] and advice from knowledge synthesis specialists [60–63] to systematically identify and map key concepts in the peer-reviewed and indexed literature. We followed a five-step scoping review methodological process previously reported in our published protocol [23].

To conceptualize remuneration and extrinsic and intrinsic incentives, we identified and categorized the factors in each category based on a literature review **(**Table 1). This approach allowed us to capture a broad range of incentives reflecting how various factors can drive both personal and collective motivations within an interprofessional team. This review will examine incentives at the individual and group/team levels. Table 1 outlines whether the incentives are focused at the individual or team level. In this context, "factors" refers to specific elements or components that contribute to or comprise remuneration, extrinsic incentives, and intrinsic incentives.

**Table 1:** Intrinsic and extrinsic incentives framework

**Intrinsic Incentives**	**Example(s)**
Autonomy [64, 65] and Empowerment (Individual Level) [66]	Decision-making authority; Control over work processes; Ownership of tasks and responsibilities [67]
Competence and Mastery [64, 66, 67] (Individual Level)	Comprehensive knowledge of skills (competent and confident); Being good and getting better at what you do (i.e., feedback, learning from experience, performance data) [66, 68] Acknowledgment of personal accomplishments [69]; Comprehensive knowledge of or skill in work; Being good and getting better at what you do [66]
Purpose, Values, and Mission Alignment [68, 70] (Individual Level)	Connection to the broader organizational mission; [70] Shared team goals [68]
Feedback [70], Recognition [70], and Performance Evaluation (Individual Level)	Regular feedback [70]; Opportunities for self-assessment and reflection
Sense of Belong and Relationships (Individual and Team Level)	Supportive [69] and collaborative atmosphere; Strong interpersonal relationships [71]; Psychological safety and trust [68]; Belonging to a team or organization; Contributing to shared goals [66]; Inclusive work environment [67, 70]; Respect for diverse perspectives and experiences; Alignment with organizational values and culture [70]; Focus on patient care and community health
Job Variety and Intellectual Challenge (Individual and Team Level)	Diverse tasks and roles [69]; Problem-solving; [70, 71] Critical thinking; Opportunities for creativity and innovation [68]
Social purpose [71] (Individual Level)	Having a positive impact on patients or colleagues. [66] Focus on patient needs and preferences [68]; Development of strong patient-provider relationships; Empathy [68] Compassion in care delivery [68]
Work-Life Balance and Well-being [64, 68] (Individual Level)	Flexible work schedules [72]; Support for stress management and self-care; [68] Resources to maintain well-being [64]
Job Satisfaction (Individual Level)	Recognition for work tasks completed; The level of responsibility; Enjoyment with work tasks; enjoyment of working conditions; Agreement with company policy, including salary [73, 74]
**Extrinsic Factors**	**Examples**
Financial incentives [76–82] (Team Level)	Pay-for-performance programs, performance-based bonuses [76–82]
Organizational culture [68, 71] (Team Level)	Leadership promoting trust [68], open communication [68], and shared decision-making[68]
Training and education [70, 71] (Team Level)	Employer-sponsored ongoing professional development programs [82]
Professional development opportunities [71, 82] (Team Level)	Employer-sponsored: 1. Access to continuing education for high-performing team members 2. Meeting performance expectations [71]
Guidelines and Protocols [67, 72] (Team Level)	Written job descriptions, protocols for specific tasks, and roles and responsibilities [72]
Communication tools and technology [67] (Team Level)	Employer investments in electronic health records, secure messaging platforms, and telehealth [67]
Access to resources [71] (Team Level)	Employer investment in resources: 1. Equipment for teams demonstrating effective collaboration 2. Staffing support for improved patient outcomes
Team meetings and huddles [68] (Team Level)	Time provided for regular meetings to discuss patient cases, review progress [68], and address challenges
Performance measurement and feedback [70, 76, 78] (Team Level)	Quality indicators, patient satisfaction surveys, and regular feedback on team performance [76, 78]

We searched for peer-reviewed literature in Medline, CINAHL, Embase, PsycINFO and EconLit (see Additional File 1). We conducted a hand search of reference lists of included studies using forward and backward citation tracking [75]. We also conducted a grey literature search of the first 100 pages of Google and Google Scholar. This page limit was set to balance thoroughness and feasibility, as it allowed for the identification of relevant themes and sources without overextending the scope of the review. After 100 pages, results typically became less pertinent, with diminishing returns in relevance and quality. This approach ensured that we captured a representative sample of grey literature without compromising the timeliness of the review. Table 2 provides inclusion and exclusion criteria. In cases where reviewers did not reach consensus on the inclusion of an article, a third independent reviewer (MA) was consulted to provide a final decision.

**Table 2:** Inclusion and exclusion criteria

**Variable **	**Description**	**Inclusion**	**Exclusion**
**Population**	Primary care team	PC teams with two more disciplines in primary care clinic or organization	Single or team practice of family physicians/general practitioners
		Teams with out-of-pocket costs by providers or through government funding	In-patient setting (e.g., acute care, rehabilitation)
**Intervention**	Remuneration	Remuneration: salary, FFS, bundled payment/global fee/case rate, P4P and capitation, blended capitation, blended salary	
		Before and after studies or comparison of PC team models	
	Extrinsic Incentives	Extrinsic Incentives: pay rises, bonuses, paid leave, annual recreational plans and professional development	
	Intrinsic Incentives	Intrinsic Incentives: autonomy, challenge and responsibility, the opportunity for advancement, perceived significance of the work and personal satisfaction	
**Outcomes**	The outcome of the included articles.	A broad range of indicators: Team (team collaboration, team effectiveness), patient outcomes (quality, safety, satisfaction), provider outcomes (satisfaction), system outcomes (cost-effectiveness, productivity and performance; emergency department visits, hospital readmissions; equity etc.)	
**Study Designs**	The study design of included articles.	Empirical studies that use quantitative, qualitative, or mixed methods	Studies that focus on theories/methods, opinion letters, commentaries, editorials, protocols, reviews (literature, systematic or scoping review)
		Pilot Studies (e.g., Feasibility or Utility studies), Action Research, Case Studies, Ethnography, Evaluation Methods, Evaluation, Research Experiments, Focus Groups, Field Studies, Interviews, Mail Surveys, Mixed Methods Research, Naturalistic Observation, Online Surveys, Participant Observation, Participatory Research, Qualitative Research, Questionnaires Research, Statistical Analysis, Statistical Studies, Telephone Surveys	
**Language**	The language of included articles.	English language	
**Time Period**	The publication time of included articles.	Time period 2000 to 2022	

A data extraction form assisted with the narrative analysis to identify common themes in the data [59]. Two team members independently charted data using the data extraction form on Covidence and reviewed by the PI [59, 60]. The charted data was condensed into summary tables based on the research questions. A quality assessment of the included studies was independently conducted by two members using the Mixed Methods Appraisal Tool (MMAT) [83]. The MMAT was chosen for its versatility and established reliability in evaluating studies across qualitative, quantitative, and mixed-methods designs, making it well-suited for the diverse methodologies represented in this review [83]. The tool comprises a series of criteria specific to each methodological category, allowing reviewers to systematically assess the quality of studies in a standardized manner [83]. Two reviewers conducted the quality assessment independently, comparing results and discussing discrepancies to reach consensus.

Given the diverse nature of the included studies and outcomes, we opted for a narrative synthesis approach to better capture the complexities across methodologies [84]. As such, statistical analyses were deemed inapplicable, which aligns with the study protocol and scoping review reporting practices where frequency counts, rather than detailed statistical analyses, are often appropriate to summarize diverse types of evidence [23, 85].

## Results

**Fig. 1:**
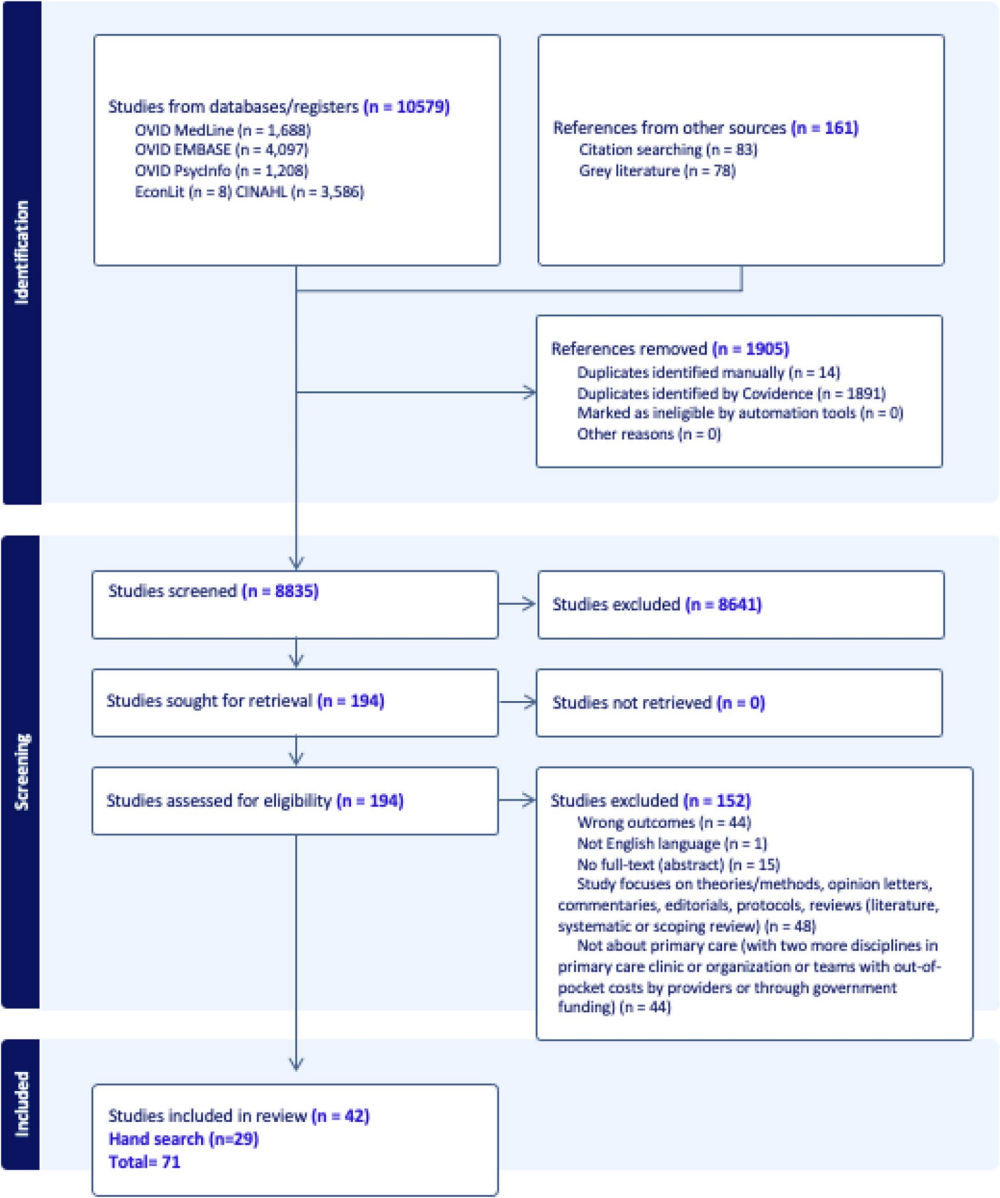
PRISMA flow diagram

The reporting of this review was informed by the Preferred Reporting Items for Systematic Reviews and Meta-Analyses extension for scoping reviews (PRISMA-ScR) [86]. Figure 1 presents the PRISMA flowchart. 8835 records were initially screened, 194 underwent a full-text review, and 42 met the inclusion criteria. A hand search yielded an additional 29 articles.

The included studies were deemed high-quality according to the MMAT assessment (Supplemental Material 1).

### Study characteristics

Table 3 provides an overview of article characteristics. The studies primarily originated from the UK (22.5%), USA (21.1%), and Canada (39.4%). Most studies employed either quantitative (37%) or qualitative (46%) study designs, with the remaining using mixed methods (17%).

**Table 3:** Article characteristics

Author, Year, Country, Study Design	Objective	Data Collection Methods	Operationalization of Team-Based Care	Healthcare Providers included in Team-Based Care	Results	Conclusions	Recommendations
Arevian, M., 2005 [87], USA, Quantitative	To evaluate the impact of collaborative practice on the quality and cost of effective care for diabetic patients in a primary health care center	Audit process, analysis of patient medical records	Primary health care centre	Team includes general practitioners, social workers, nurses, public health officer, dietician, specialists	The results indicated a high level of enthusiasm, support and the development of team spirit at the process level. At the outcome level there was improvement in documentation, increase in patient recruitment, increase in continuity of care, improvement of glycemic control and decreased cost	Collaborative practice interventions improved process and outcome variables for diabetic patients	It is suggested that this model could be developed for use in the care of other chronic diseases Healthcare teams can benefit from improvements in documentation in order to increase patient recruitment Further work is required to explore the impact on quality of patients’ life and satisfaction with the program
Bareil et al., 2015 [88], Canada, Qualitative	To better understand the driving forces during the early stage of the implementation process of a community-driven and patient-focused program in primary care titled “TRANSforming Interprofessional cardiovascular disease prevention in primary care” (TRANSIT)	Focus groups, interviews	Members of the primary care community	Various decision makers, family physicians, nurses, nutritionists, pharmacists, and others	The analyses revealed three key forces that facilitated the implementation of interprofessional collaborative practices in primary care: 1. Opportunity for dialogue through the Interprofessional Facilitation Team (IFT) 2. Active role of the External Facilitator (EF) 3. Change implementation budgets	This community-based and patient centered study aimed to implement an interprofessional intervention program in primary care. Interim findings of this qualitative evaluation highlight a trilogy of driving forces: an interprofessional team and IFT, an EF, and change implementation budgets. The three forces should be activated simultaneously because they strengthen one another. Interventions based on interprofessional collaboration in a context in which primary care is undergoing transformation often have proven to be challenging	To address this challenge, change managers should activate an opportunity for dialogue; include the active role of the external facilitator, and change implementation budgets to enhance the implementation process
Beaulieu et al., 2013, Canada, Mixed Methods [89]	To identify the organizational characteristics of primary care practices that provide high-quality primary care	Cross-sectional observational study. The study used the Organizational Questionnaire, and the Team Climate Inventory to measure Team process	Primary care practices	Primary care practices, patients	Findings suggest high-quality care can be achieved by practices with different organizational models. The authors identified organizational factors that, beyond models, can improve care. As hypothesized, organizational contribution to technical quality differed according to the nature of care considered	The study identified a common set of organizational characteristics associated with high-quality primary care (e.g., effectiveness of team process, presence of competence-maintenance mechanisms, organizational access). Most of these characteristics are amenable to change, through either health policy or practice-level organizational changes	Beyond investing in new delivery models, decision-makers should invest in helping primary care practices reach a high level of functioning by fostering group practice and effective team-based care
Burgess et al., 2011 [90], Canada, Qualitative	To explore the meaning of nurse practitioner (NP) role integration to develop a framework	Participatory action research whereby journal articles were shared and discussion of NPs’ patterns of everyday practice, experiences of role development, and factors contributing to collaboration and role integration were explored	Community Health Centre	NPs, physician	The study uncovered 5 dimensions of NP role integration: autonomy, recognition, inclusion, contribution, and alliance	The framework is beneficial for policy leaders, decision-makers, and researchers as it helps them overcome obstacles related to integrating roles, assess the effectiveness of roles, and ensure the safety and protection of the NP role	The framework can be refined and used for policy leaders, decision-makers, and researchers to determine the status of NP role integration within a health-care setting or area and to identify deficiencies and strategies for role advancements
Campbell et al., 2008 [91], UK, Qualitative	To conduct an in-depth exploration of family physicians' and nurses' beliefs and concerns about changes to the family health care service as a result of the new pay-for-performance scheme in the United Kingdom (Quality and Outcomes Framework [QOF])	Semi-structured Interviews	General Practice	Family doctors, nurses	Participants believed the financial incentives were sufficient to change behavior and to achieve targets. The findings suggest that it is not necessary to align targets to professional priorities and values to obtain behavior change, although doing so enhances enthusiasm and understanding. Participants agreed that the aims of the pay-for-performance scheme had been met in terms of improvements in disease-specific processes of patient care and physician income, as well as improved data capture. It also led to unintended effects, such as the emergence of a dual QOF-patient agenda within consultations, potential deskilling of doctors as a result of the enhanced role for nurses in managing long-term conditions, a decline in personal/relational continuity of care between doctors and patients, resentment by team members not benefiting financially from payments, and concerns about an ongoing culture of performance monitoring in the United Kingdom	The QOF scheme may have achieved its declared objectives of improving disease-specific processes of patient care through the achievement of clinical and organizational targets and increased physician income, but the findings suggest that it has changed the dynamic between doctors and nurses and the nature of the practitioner-patient consultation	Further research is needed to observe the evolution and multiple effects of this dynamic pay-for-performance scheme
Campbell et al., 2010 [92], UK, Quantitative	To examine patient reports of quality of care between 2003 and 2007	Questionnaires	General practice	General practices, patients	There were no significant changes in quality of care reported by the study sample between 2003 and 2007 for communication, nursing care, coordination, and overall satisfaction. The findings also suggest that patients in the United Kingdom are significantly less likely to report being able to make an appointment with their usual physician, and they report lower ratings of continuity of care in 2007 compared with 2003. This finding was observed in patients with chronic illness and in population samples of patients. It may not be surprising that continuity has decreased when initiatives to improve access to physicians have been prioritized	There is relative improvement in access to care for patients with chronic illnesses, but all patients from the study noted finding it harder to obtain continuity of care. This outcome can be related to the incentives to provide rapid appointments for patients or to the increased number of specialized clinics in primary care	The possibility of unintended effects needs to be considered when introducing pay for performance schemes
Campbell et al., 2011 [93], UK, Qualitative	To explore GP and practice staff views and experiences of exception reporting in the Quality and Outcomes Framework (QOF)	Semi-structured Interviews	General Practice	General practitioners, 20 practice managers, 13 practice nurses, and nine other staff	Three key themes emerged in the data related to exception reporting: •reasons for exception reporting; •the level and appropriateness of exception reporting; and • the threat of external scrutiny on behaviour. This study suggests that practise staff view exception reporting as a crucial and clinically essential component of the QOF. Exempting patients was typically regarded as a "exception to the rule," and improper exempting was frequently carried out by "other" practises.When exception reporting was used, it was either justified in terms of providing patient-centered care within a framework of population-based health measures or because the indicators' poor face validity for specific patients	Exception reporting is seen by most GPs and practice staff as an important and defensible safeguard against inappropriate treatment or over-treatment of patients. However, a minority of practitioners also saw it as a gaming mechanism	The majority of practises would benefit from employing exception reporting as a clinical safeguard to high-quality, individualised patient care within a flexible, evidence-based framework
Cashman et al., 2004 [94], UK, Qualitative	To foster and guide the development of interdisciplinary healthcare teams towards a collaborative, integrated approach to care delivery, and to evaluate the effectiveness of this approach by assessing values known to reflect effective team functioning	Questionnaire survey called "The System for the Multiple Level Observation of Groups (SYMLOG)" was used	Community Health Centre	Family practice physicians, nurse practitioners, and physician assistants	Properties include (a) the heterogeneity of team composition, (b) role conflict and role overload, (c) constraints placed on members by the larger organizational structure, and (d) members' lack of knowledge about the process of team development. These properties are considered generic and widely applicable	Team members' objective assessments, as well as their lived experiences, provide detailed reaffirmation that, in order to sustain effective team functioning, organisational structures and reward systems must be aligned so that they can support the team's vision and goals, is particularly significant in this study	Intentional team training and development, combined with dedicated time for team meetings, can result in team members expressing values consistent with high functioning teams Methods for reducing team turnover are also required to ensure that interdisciplinary teams grow
Cassou et al., 2020 [95], France, Quantitative	To explore the overall effect of practicing in multiprofessionalprimary care groups (MPCG) on GPs’ income in the context of the French reform in which GPs are considered the pillar of any MPCG. To this aim, the authors analyze the impact of MPCGs on GPs’ medical activity in terms of both the quantity of medical services and the number of patients seen, to highlight the organizational features of MPCGs and their impact with respect to FFS and capitation payments	Administrative database that combined National Health Insurance data	Multi-Professional Primary Care Groups (MPCGs)	General practitioners (GPs), nurses, pharmacist, dental surgeons, specialist, other care providers (unspecified)	The study found that General Practitioners (GPs) enrolled in MPCGs experienced an increase in income 2.5% higher than that of other GPs during the period studied. Moreover, these GPs saw a greater increase in the number of patients (88 more) without involving a greater increase in the quantity of medical services provided. A complementary cross-sectional analysis for 2014 revealed that these changes were not detrimental to quality in terms of bonuses related to the French pay-for-performance program for that year	The results suggest that labor and income concerns should not be a barrier to the development of MPCGs, and that MPCGs may improve patient access to primary care services. A greater increase in the number of patients seen by the GPs’, were not detrimental to quality in terms of bonuses related to the French pay-for-performance program for the year 2014	Support the organizational properties of teamwork to help GPs in MPCGs to see and follow more patients without increasing the quantity of their delivered services. Policymakers should support MPCGs as it increases GPs’ ability to treat a larger number of patients without increasing the quantity of services
Delva et al., 2008 [96], Canada, Qualitative	To explore the views of members of primary health care teams regarding what constitutes a team, team effectiveness and the factors that affect team effectiveness in primary care	Focus groups; Surveys (Team Survey by Delva and Jamieson, 2006)	Academic Clinical Setting	Members of the Department of Family Medicine at Queen’s university. Residents, secretaries, float/replacement nurses, nutritionists, social workers, and administrative staff varied among these teams. Two teams were uni-professional: an administrative/management team (four members) and a nursing team (nine members)	Twelve themes were identified that related to the impact of dual goals/obligations of education and clinical/patient practice on team relationships and learners; the challenges of determining team membership including non-attendance of allied health professionals except nurses; and facilitators and barriers to effective team function	Cultural shifts in primary care that embrace all team members (i.e., professional and support staff) and learners will be important if interprofessional teamwork is to be modeled and learned in academic practice settings	Further research based on modern concepts of complex adaptive systems is needed to determine how best to support the changes needed to implement effective teamwork in primary care
Dieleman et al., 2004 [97], Canada, Qualitative	To examine the perceptions of pharmacists, physicians and nurses as they worked together in community-based teams to provide care to 199 high-risk community dwelling individuals	Pre- and Post- test design; Questionnaires	Community-based health center	Nurses, Pharmacists, Practitioners	The results indicate that the providers found that working in a team environment was very useful when they dealt with complex primary-care patients. The study results also show that providers relied on their team members for support, after learning the various skills and knowledge offered by each team member. Communication is also a significant aspect of team effectiveness, as it played a role in the overall satisfaction of the team. The results are aligned with previous studies that found collaborative workspaces positively impact job satisfaction	This study concludes by stating that empirical information about community teams requires research, specifically examining the importance of open communication, respect, and understanding the expertise of other members	NR
Dimitrovova et al., 2020 [98], Portugal, Quantitative	To evaluate the impact of the Family Health Units (FHUs) implementation on population health outcomes, measured by the rate of hospitalizations for ambulatory care sensitive conditions (ACSC), i.e. avoidable hospital inpatient admissions, and to explore the effectiveness of the pay-for-performance in primary care by analysing the subset of disease specific hospitalizations for ACSC related to the financial incentives	Portuguese Central Administration of the Health System and the National Institute for Statistics	Family Health Units (FHU)	General practitioners, nurses, administrative technicians	The results showed that there were no statistically significant changes in disease-specific hospitalization rates as a result of the implementation of the FHUs targeted by the pay for perforamce (P4P). There was no significant impact of FHU implementation on the reduction of ACSC hospitalization rates, including the ACSC-incentivized hospitalizations. The only statistically significant effect of FHU implementation was a reduction in the rate of urinary tract infection ACSC, which was a non-incentivized area	No significant impact of the FHUs implementation on the reduction of the hospitalization rate for ACSC was found. This result also held for conditions specifically incentivized by the P4P scheme. This finding, questions the capacity of P4P payment mechanism to achieve better health outcomes, and invites a more careful and evidence-based action toward its wider diffusion	Decision-makers should be cautious when assuming payment mechanisms will achieve better health outcomes
Doran et al., 2006, UK [99], Quantitative	To examine the performance of family practices in England in the first year of the pay-for-performance program between April 2004 – March 2005	Data analysis of practice performance on the clinical indicators operated by the Natioal Health Service(NHS) information centre; United Kingdom census	Family Practices	Patients, Family Practice physicians	Results indicate in the first year of the pay-for-performance program, English family practitioners performed extremely well with respect to the quality targets, which explains that financial incentives affect physician behavior	The United Kingdom experience suggests that greater changes in professional practice can be achieved through pay-for-performance programs	Financial incentives should be aligned to physicians' professional values to avoid serious distortions of care
Doran et al., 2008 [100], UK, Quantitative	To examine the relation between socioeconomic inequalities and delivered quality of clinical care in the first 3 years of the quality and outcomes framework financial incentive scheme	Data analysis of practice performance on the clinical indicators operated by the NHS information centre; United Kingdom census	General Practices	General practitioners, Patients	Results suggest that financial incentive schemes have the potential to make a substantial contribution to the reduction of inequalities, improvement of care delivery of clinical care related to area deprivation	Generation of more equitable provision of prevention and care for these disorders means that the use of financial incentives seems to have the potential to make a substantial contribution to the reduction of health inequalities	NR
Doran et al., 2010, UK [101], Quantitative	To describe the comparative performance of small practices on the United Kingdom's pay-for-performance scheme, the Quality and Outcomes Framework (QOF)	Longitudinal analysis	Family Practice	Family physicians, patients	Aspects of quality are associated with smaller practices, such as patient ratings of access or continuity of care, while others are associated with larger practices, such as data recording or organization of services. However, it's important to note that there is no consistent association between practice size and differences in outcomes	The effect of the pay-for-performance scheme appears to have been to reduce variation in performance, and to reduce the difference between large and small practices	NR
Drew et al., 2010 [102], Canada, Mixed Methods	To explore the level of perceived team effectiveness in primary care networks (PCNs) within three health regions in Alberta, Canada as determined by the Team Effectiveness Tool (TET). A secondary exploratory objective was to identify strategies, including team composition, that relate to team effectiveness in the PCNs	Semi-structured questionnaire study design, using the Team Effectiveness Tool (TET)	Primary Care Networks	Physicians, registered nurses, licensed practical nurses, physical therapists, administrative-related positions, and team members with mixed designations	The results identified strategies related to regular meetings/communication, team development and, to a lesser degree, purpose/goals identified as helpful in developing team effectiveness. Leadership was not highlighted; instead, frequent regular meetings was consistently identified, as were innovation in service delivery and role clarification	Findings suggest a need for strategies to focus on regular and frequent meetings as a communication tool in the primary care team setting. Additionally, the areas of relative weakness – team partnership, team purpose and vision and team roles – might benefit from growth. A redistribution of resources (time, money, energy) to these areas might help teams become better rounded. In particular, team partnership is clearly an area of weakness among the teams studied, and it might be strategic for PCNs to prioritize addressing and bolstering this component	Explore the application of the TET instrument in the process of developing some standardized evaluation for PCNs
Drummond et al., 2012 [103], Canada, Mixed Methods	To explore the status and processes of interprofessional work environments and the implications for interprofessional education in a sample of family medicine teaching clinics	Semi structured focus group interviews using a purposive sampling procedure	Academic family medicine clinics	7 family physicians, 1 registered nurses, 5 licensed practical nurses, 2 residents, 1 psychologist, 1 informatics specialist, 1 pharmacist, 1 dietitian, 1 nurse practitioner, 1 receptionist, and 1 respiratory therapist	The study suggests that having leaders who prioritize interprofessional collaborative clinical work is crucial for the growth and continuity of interprofessional practices and the related interprofessional education	The study concluded that the existence of clear and explicit leadership towards interprofessional work and education was the key factor in the implementation of interprofessional work in primary care. The study suggested that there is substantial scope for improvement in the organization, conduct, and promotion of interprofessional education for Canadian primary care	Primary care teams should implement clear and explicit leadership
Gemmell et al., 2009 [104], UK, Quantitative	To describe changes in practice team size and composition, and the workload of doctors and nursing staff, before (2003) and after (2005) the introduction of the pay-for-performance contract for general practice	Practice profile questionnaires and staff workload diaries	General Practice	Doctors, nursing staff	The findings suggest that expanding nursing staff roles may be an effective strategy for increasing the quality of primary care. The number of practice staff increased with greater increases observed for nursing staff than doctors. There was no change in the average number of hours worked per week by nursing staff or doctors but nurse visit rates increased while doctors' rates decreased	General practices may have responded to the 2004 contract by increasing staffing levels, with nursing staff absorbing a higher proportion of the clinical workload and doctors focusing more attention on chronic and preventive care	Expanding nursing staff roles may increase the quality of primary care but may lead also to intensification of nurses' work
Gene-Badia et al., 2007 [105], Spain, Quantitative	To assess whether the implementation of these economic incentive schemes has had an impact on the quality of professional life (QPL) of both physicians and nurses and on end-user satisfaction	Before-after study	Primary Care Teams	Physicians, nurses	The results show that there is a relationship between the implementation of economical incentives and changes in the Quality and Productivity Level (QPL) of health personnel, as well as end-user satisfaction. The introduction of economical incentives can incentivize health personnel to improve their performance and meet quality targets, leading to positive changes in the QPL. This, in turn, can impact end-user satisfaction, as improved quality of care is likely to result in higher satisfaction among patients or service users. The specific nature and magnitude of these relationships may vary depending on the specific context and implementation of the incentives	Incentives related to quality of care annual targets may increase physicians' perception of burden and it may have a negative impact on consumer satisfaction. Incentives on long-term professional development seem to be related to an increase in professionals' perception of support from the management structure. Among nurses, this increase is related to an improvement of user satisfaction	Analyze professional incentives impact on relevant outcome measures before spreading such reforms to a broad amount of professionals
Glazier et al., 2016 [106], Canada, Quantitative	To compare outcomes of family Health Teams (FHT) patients in relation to other major models of primary care in Ontario, over time	Administrative datasets, FHT demographics	FHT, Community Health Center (CHC)	Physicians, patients	FHT and other capitation-based models have somewhat wealthier and healthier populations than other models of care. Given that physicians had a free choice of models, these patterns likely reflect the way that payment incentives such as capitation and bonuses favour certain types of practices	FHT generally performed well in cancer screening and diabetes care, with improvements over time that were larger than those of fee-for-service models but not consistently better than other capitation models. Improvements over time in cancer screening in FHTs were not consistently better than in CHCs	The findings about FHT trends over time should be placed in the context of the work performed by the Conference Board of Canada in its FHT evaluation
Glazier et al., 2012 [39], Canada, Quantitative	To characterize primary care models in Ontario by demographics, practice location and case mix and to examine emergency department (ED) use by patients/clients in each model before and after controlling for their characteristics	CHC data, the Registered Persons Database; physician billings from the Ontario Health Insurance Plan; hospital Discharge Abstract Database; ED visits from the National Ambulatory Care Reporting System; the Ontario Drug Benefit Program; Client Agency Program Enrolment tables, the Rurality Index of Ontario for urban–rural residence, and 2006 Census of Canada data for sociodemographic variables	Community Health Centres, Family Health Groups, Family Health Networks, Family Health Organizations	Physician, nurse practitioner, and other non-physician providers	Physician, nurse practitioner, and other non-physician providers	Ontario’s primary care models serve different populations and are associated with different outcomes. A move away from fee-for-service reimbursement may be desirable for a high functioning health care system, but how alternate payment mechanisms are structured appears to matter a great deal. The largest current models of care have been costly but have had limited impact on population access to care, which was a key aim. The capitation and team models that have received the most resources are looking after relatively advantaged groups and are associated with higher than expected ED visits	The existing bonus payment aimed at discouraging the utilization of emergency medicine services appears to be ineffective in achieving its intended purpose and can be revisited. Modify capitation rates to consider healthcare needs, with the objective of attracting a greater number of high-needs patients and practices to participate in these models
Goldman et al., 2010 [107], Canada, Qualitative	To examine the perspectives and experiences of family health team (FHT) members regarding interprofessional collaboration and perceived benefits	Semi structured interviews	Family Health Teams	Family physicians, nurse practitioners and nurses, pharmacists, managers, social workers, and dietitians	The study identified the essential role of the FHT manager and physician leadership in supporting and sustaining an interprofessional FHT. The physical layout of the FHT's central practice space was another important factor that can promote or inhibit interprofessional collaboration. The study documented the different strategies and initiatives being used by the FHTs to support interprofessional care, which can be categorized into organizational, practice-based, and educational interventions. Participants perceived that FHTs were progressing toward an interprofessional approach to delivering care, which was making positive changes in patient care, but further evaluation is required to understand the relationship to the realities of accessibility of care and improvement in patient health outcomes	The study concluded that effective team-based primary care requires addressing issues such as roles and scopes of practice, leadership, and space, and provided a framework for understanding different types of interprofessional interventions used to support interprofessional collaboration	Supporting roles and scopes of practice, leadership, and space to contribute to effective team-based primary care
Grant et al., 2009 [108], UK, Mixed Methods	To report the impact of the new 2004 General Medical Services (nGMS) contract which prioritizes the ‘Quality and Outcomes Framework’ (QOF), and the financial incentives contained within it on professional boundaries in United Kingdom general practice	Field notes (ethnography)	General Practice Clinics	General Practitioners, Nurses	The four practices in this study illustrate the complexity of recent changes taking place in UK general practice through the financial incentives embedded in the QOF. The most significant change is the way in which practices have created internal QOF teams that cut across traditional clinical and administrative hierarchies and boundaries. These were not as clearly contested by participants as the changes that were taking place at the more established clinical boundaries, which were readily accounted for through the use of existing rhetorical strategies	The creation of new managerial roles through the QOF has reinforced and significantly extended an existing trend towards ‘bureaucratization’ and professional restratification within general practice, with QOF teams drawn from a range of disciplines	NR
Greene et al., 2014 [109], USA, Mixed Methods	To examine primary care providers’ (PCPs) perception of the early impact of the compensation model on practice and satisfaction	In-Depth Interviews and online survey	Primary care clinics	Family medicine physicians, internist, pediatrician, nurse practitioners, and physician assistant	The team-based, quality-focused compensation model was effective in improving the quality of care and patient outcomes. The compensation model encouraged collaboration and teamwork among providers, which led to improved patient care. The model incentivized providers to focus on preventive care and chronic disease management, which resulted in better health outcomes for patients. The compensation model was financially sustainable and cost-effective, as it reduced the need for costly hospitalizations and emergency room visits. The comprehensive change did, however, result in lower fee-for-service billing and reductions in PCP satisfaction	PCP participation in design of compensation model is important. Prepare PCPs for the challenges of compensation at the team level. Minimize complexity and changes in the model. Transparency in PCPs’ quality performance can be powerful motivator. Do not let the compensation model get ahead of the revenue stream	Provide opportunities for ongoing feedback from PCPs Provide training, tools, and support to help prepare PCP for organizational changes, and help them with the skills they will need to collaborate
Hämel et al., 2017 [110], Germany, Qualitative	To take a closer look at possibilities of cooperation between GPs and nurses in primary care in Slovenia and Spain in the context of their country-specific primary care concepts and further refinement. The comparative analysis enabled us to identify differences in conceptual and procedural methods employed in the two countries	Background review of primary care concepts (literature analysis, expert interviews)	Primary care team	Physicians and nurses	The introduction of a new cross-professional primary care concept has integrated advanced practice nurses into general practice. Conventional hierarchies still exist, but a shared vision of preventive care is gradually strengthening attitudes towards team-oriented care. Formal regulations or incentives for teamwork have yet to be implemented. In Spain, health centres were established along with a team-based care concept that encompasses close physician–nurse collaboration and an autonomous role for nurses in the care process. Nurses collaborate with general practitioners on more equal terms with conflicts centring on professional disagreements. Team development structures and financial incentives for team achievements have been implemented, encouraging teams to generate their own strategies to improve teamwork. Furthermore, collaboration results in increased accessibility to a diverse range of expertise and skills among all team members, ultimately benefiting patient care	Team development structures and financial incentives for team achievements have been implemented, encouraging teams to generate their own strategies to improve teamwork	Invest in clearly defined structures, shared visions of care and team development
Harris et al., 2016 [111], Canada, Australia, USA, Qualitative	To assess the impact of reform policies and interventions that have aimed to create or enhance teamwork on professional communication relationships, roles, and work satisfaction in primary health care (PHC) practices	Synthesis and secondary analysis	Primary Care Practices	Primary care teams	The results show a diverse range of complex reforms seeking to foster interprofessional teamwork in the care of patients with chronic disease. The impact on communication and relationships between different professional groups, the roles of nursing and allied health services, and the expressed satisfaction of PHC providers with their work varied more within than between jurisdictions. These variations were associated with local contextual factors such as the size, power dynamics, leadership, and physical environment of the practice. Unintended consequences included deterioration of the work satisfaction of some team members and conflict between medical and nonmedical professional groups	The variation in impacts can be understood to have arisen from the complexity of interprofessional dynamics at the practice level. The same characteristic could have both positive and negative influence on different aspects (eg, larger practice may have less capacity for adoption but more capacity to support interprofessional practice). Thus, the impacts are not entirely predictable and need to be monitored, so that interventions can be adapted at the local level	Policymakers need to be aware of the complexity of the PHC context into which reforms are introduced and the consequent variation in impacts and responses.
Hepp et al., 2014 [112], Canada, Qualitative	To examine organizational factors influencing the functioning of inter-professional teams in select primary care networks (PCNs) in Alberta	Face-to-face and telephonic interviews	Primary Care Networks	Team includes; dietitian, exercise specialists, manager, registered nurse, mental health clinician, pharmacist, physician, physiotherapist, resource navigator, social worker, office assistant etc	The study highlights that physical infrastructure was a barrier to team functioning, especially inadequate space negatively affecting relationship building, collaboration, and access to expertise. Co-location of team members in a PCN office facilitated collaboration, communication, and relationship building, but impeded day-to-day interactions between interprofessional team members and physicians. Decentralization allowed relationships and trust to build between physicians and team members working together in physicians' clinics but communicating with busy physicians was a challenge at these sites due to time constraints or limited private space	This study focuses on some of the organisational factors that influence team performance, such as leadership and managerial decision-making. Although researching these factors is a step forward in understanding teams from an organisational standpoint, more research on outcomes is required to fully understand organisational strategies and their impact on interprofessional teams and patient care	Create an interdisciplinary management team. Engage all stakeholders during development to identify service grants. Foster a culture of respect, continuous learning and improvement. Allow co-location Acknowledge the good work staff do. Develop an integrated EMR system between and across physician clinics and PCN offices. Orient new employees and provide ongoing education. Promote physician buy-in and support by compensating physicians for time meeting. Provide education and offering shadowing days where physicians observe interprofessional professional teams in action.
Khan et al., 2022 [113], Canada, Quantitative	To examine interprofessional teamwork within primary care practices (Family Health Teams [FHT] and Community Health Centers [CHC]) in Ontario and to investigate team-level and organisational factors related to interprofessional teamwork	The study used the Collaborative Practice Assessment Tool (CPAT) to assess the extent of interprofessional teamwork within participating primary care practices. A team profile survey was used to assess organizational characteristics	CHC and FHTs	A wide range of professions including family physicians, nurse practitioners, registered nurses, social workers, and dieticians/nutritionists, occupational therapists, chiropodists, physiotherapists, chiropractors, pharmacists, health promoters, and personal support workers	The study indicated that there were statistically significant differences in CPAT scores (a measure of interprofessional teamwork) between primary care models, with Family Health Teams (FHTs) having lower CPAT scores compared to Community Health Centers (CHCs). Using diverse communication mechanisms to share information, increasing quality improvement capacities, and age of practice, had a statistically significant positive association with CPAT scores. Increasing team size, using centralized administrative processes, a high level of information exchange, and having a mixed governance board were significantly negatively associated with CPAT score	There are several factors that may need to be addressed to support and enhance interprofessional teamwork among healthcare providers	The transition from physician-led model to a team-based lens requires comprehensive and ongoing training on interprofessional teamwork and dedicated education for team members prior to and even after joining the FHT
Khazei et al., 2020 [114], Canada, Quantitative	To assess self-rated team climate, intrinsic motivation, and burnout of a multidisciplinary team at an urgent primary care center and to explore potential relationships between the concepts	Survey administration	Multidisciplinary teams striving to achieve the Quadruple Aim	Physicians (general practitioners, family medicine physicians, and emergency physicians), nurse practitioners, registered nurses, patient care coordinators, radiology technologists, medical office assistants, laboratory assistants, pharmacists, and mental health and substance-use clinicians	The survey findings indicate a relatively high-performing multidisciplinary team, with high scores in all categories related to team climate and intrinsic motivation. Only 8% of 25 respondents met the threshold level of burnout, with no respondents indicating severe or complete burnout. Reliability analysis produced α coefficients of 0.956 and 0.945 for team climate and intrinsic motivation, respectively, indicating satisfactory reliability	The study fills a gap in the health services research literature pertaining to the performance of multidisciplinary teams	Future research is needed that focuses on the survey tool developed in this study across various organizational settings and context. Rather than focusing on extrinsic factors, the focus should be intrinsic motivation as it relates to, team climate, and burnout
Kiran et al., 2012 [115], Canada, Quantitative	To assess diabetes incentive code introduced for primary care physicians in Ontario, Canada, in 2002 on quality of diabetes care at the population and patient level	Administrative database: Ontario Diabetes Database (ODD)	Diabetes incentive codes/model	Endocrinologist or general, primary care physician visits	One-quarter of Ontarians with diabetes had an incentive code billed by their physician. The proportion receiving the optimal number of all three monitoring tests (HbA1c, cholesterol, and eye tests) rose gradually from 16% in 2000 to 27% in 2008. Individuals who were younger, lived in rural areas, were not enrolled in a primary care model, or had a mental illness were less likely to receive all three recommended tests. Patients with higher numbers of incentive code billings in 2006–2008 were more likely to receive recommended testing but also were more likely to have received the highest level of recommended testing prior to introduction of the incentive code	The shift to capitation payment and the addition of team-based care in Ontario were associated with moderate improvements in processes related to diabetes care, but the effects on cancer screening were less clear	Financial incentives may be a useful tool for improving diabetes care quality, but social and structural factors that affect health outcomes need to be explored
Kirschner et al., 2013 [116], Netherlands, Quantitative	To assess changes in performance after introducing a participatory pay-for-performance (P4P) program	Pre- and Post-measurement	General practices	General practitioners, patients	Introduction of a participatory P4P program yielded significant improvements in care delivery. Clinical care indicators, pertaining to both process and outcome	A participatory P4P program might stimulate quality improvement in clinical care and improve patient experiences with general practitioner functioning and the organization of care	More studies are needed in which the appraisal and reimbursement are based on drivers taken from behavioural economics
LaMothe et al., 2021 [117], USA, Mixed Methods	To describe the facilitators and barriers of Interprofessional Collaborative Practice (IPCP) implementation in rural clinics and the impact on decision-making and safety culture	Survey administration and Qualitative Interviews	Interprofessional Collaborative Practices	Provider (medical doctor or nurse practitioner), a registered nurse, psychologist or social worker, and other clinical staff	Significant improvement in the Global Amount of Collaboration made over time. Barriers to IPCP included high turnover, hierarchical culture, lack of role clarity, competing time demands, limited readiness for change, and physical space limitations. Facilitators included structured huddles, alignment of IPCP with organizational goals, and academic-practice partnership	The study highlighted the need and appreciation for ongoing support and guidance for team development and reinforcement. Leveraging the resources of the academic-practice partnership was key to the success of the project	Future research should examine the impact of facilitated approaches to support interdisciplinary teamwork and collaboration competencies
Lanham et al., 2009 [118], USA, Qualitative	To understand the characteristics of relationships within primary care practices	Data analyzed from National Institutes of Health (NIH)-funded studies; Observation of practices during work activities and of patient-clinician interactions; In-depth interviews with physicians and other key staff members; surveys; structured checklists	Primary care practice	Physicians, healthcare staff	Trust, mindfulness, heedfulness, respectful interaction, diversity, social/task relatedness, and rich/lean communication were identified as important in practice improvement. A model of practice relationships was developed to describe how these characteristics work together and interact with reflection, sensemaking, and learning to influence practice-level quality outcomes	Although this model of practice relationships was developed from data collected in primary care practices, which differ from other health care organizations (HCOs) in some important ways, the ideas that quality is emergent and that relationships influence quality of care are universally important for all HCOs and all medical specialties	NR
Lehtovuori et al., 2015 [119], Finland, Quantitative	To examine whether it is possible to improve clinical practice by increasing the recording of diagnoses using financial incentives to all disciplines in the care team (e.g. group bonuses)	The data was specifically derived from the electrical patient chart system (Tieto LTD, Helsinki, Finland)	Municipal health service teams	There were 6–8 doctors and 6–8 nurses per team	The proportion of doctor visits having recorded diagnoses in the teams was about 55% before starting to use group bonuses and 90% after this intervention. There was no such increase in control units. The effect of the intervention weakened slightly after cessation of the group bonuses	Group bonuses may provide a method to improve clinical practices in primary care. Yet the putative desired effects obtained with these financial incentives may slowly start to erode if these bonuses are withdrawn	Group bonuses may provide a method to improve clinical practices in primary care
Lester et al., 2013, UK [120], Qualitative	To obtain a longer-term perspective on the implementation of the Quality and Outcomes Framework (QOF) from General Practitioners and primary healthcare teams before memories of working in a pre-pay-for-performance era became less reliable	Semi-structured interviews	Healthcare practices, and General practice clinics	General Practitioners (GPs)	Pay for performance is accepted as a routine part of primary care in England, with previous more individualistic and less structured ways of working seen as poor practice. The size of the QOF and the evidence-based nature of the indicators are regarded as key to its success. However, pay for performance may have had a negative impact on some aspects of medical professionalism, such as clinical autonomy, and led a significant minority of GPs to prioritise their own pay rather than patients' best interests	Pay for performance indicators are now welcomed by primary healthcare teams and GPs across generations. Almost all interviewees wanted to see a greater emphasis on involving front line practice teams in developing indicators. However, almost all GPs and practice managers described a sense of decreased clinical autonomy and loss of professionalism	Calibrating the appropriate level of clinical autonomy is critical if pay for performance schemes are to have maximal impact on patient care
MacNaughton et al., 2013 [121], Canada, Qualitative	To explore how roles are constructed within interprofessional health care teams, including the different types of role boundaries, the influences on role construction, and the implications for professionals and patients	The data collection included interviews and non-participant observation of team meetings	Primary health care teams	Clinical director, manager, nurse practitioners, physician, registered nurses and registered practical nurse), pharmacist, dietician, social worker, mental health counselor, chiropodist, laboratory technician, administrative assistant	The study found that role boundaries can be organized around interprofessional interactions (autonomous or collaborative roles) as well as the distribution of tasks (interchangeable or differentiated roles). Different influences on role construction were identified, including structural, interpersonal, and individual dynamics. The study also found that empowering team members to develop autonomy can enhance collaborative interactions, while more interchangeable roles could increase the potential for power struggles	The study identified three categories of influences on role construction in interprofessional health care teams: structural factors related to the workplace, interpersonal factors such as trust and leadership among team members, and individual dynamics including personal attributes. The implications of role construction included professional satisfaction and improved wait times for patients	Develop strategies for empowering team members to develop autonomy can enhance collaborative interactions
Maisey et al., 2008 [122], UK, Qualitative	To understand the effects of a large scale 'payment for performance' scheme (the Quality and Outcomes Framework [QOF]) on professional roles and the delivery of primary care in the English National Health Service	Semi structured interviews	General practice	24 Clinicians, 1 general practitioner, 1 practice nurse	Participants reported substantial improvements in teamwork and in the organization, consistency and recording of care for conditions incentivized in the scheme, but not for non-incentivized conditions. The need to carry out and record specific clinical activities was felt to have changed the emphasis from 'patient led' consultations and listening to patients' concerns. Loss of continuity of care and of patient choice were described. Nurses experienced increased workload but enjoyed more autonomy and job satisfaction. Doctors acknowledged improved disease management and teamwork but expressed unease about 'box-ticking' and increased demands of team supervision, despite better terms and conditions. Doctors were less motivated to achieve performance indicators where they disputed the evidence on which they were based. Participants expressed little engagement with results of patient surveys or patient involvement initiatives. Some participants described data manipulation to maximize practice income. Many felt overwhelmed by the flow of policy initiatives	Payment for performance is driving major changes in the roles and organization of English primary health care teams. Non-incentivized activities and patients' concerns may receive less clinical attention	Practitioners would benefit from improved dissemination of the evidence justifying the inclusion of new performance indicators in the QOF
Markon et al., 2017 [123], Canada, Quantitative	To estimate the associations and predictive relationships among these variables and to test, through structural equation modelling, whether the data fit the theoretical model formulated under the Input-Mediator-Outcome-Input framework and correspond with existing literature on the variables of interest	Questionnaires administered	Local health service networks (being a member of a public mental health specialised care or primary care team comprising at least three members from two or more professions)	Front-line practitioners (e.g. general physicians, social workers, and nurses) and specialists (e.g. psychiatrists)	The structural equation model provided a good fit for the data and explained 51% of the variance of work role performance. Perceived collaboration, and confidence in the advantages of interprofessional collaboration, involvement in the decision processes, knowledge sharing, and satisfaction with the nature of the work partially mediated the effect of perceived interdependence among team members on work role performance. Therefore, perceived interdependence among team members had a positive impact on the work role performance of mental health care professionals mostly through its effect on favourable team functioning features	Increased interdependence of mental healthcare professionals would be more likely to enhance work role performance if team-based interventions promote collaborative work and interprofessional teaching and training programs are jointly implemented. Participation in the decision process and knowledge sharing should also be fostered, by adopting knowledge management best practices	Healthcare managers should promote collaborative work, knowledge sharing, and participation in decision-making
McDonald et al., 2007 [52], UK, Qualitative	To explore the impact of financial incentives for quality of care on practice organisation, clinical autonomy, and internal motivation of doctors and nurses working in primary care	Ethnography (field notes); interviews	General Practice	12 general practitioners, nine nurses, four healthcare assistants, and four administrative staff	Three major themes emerged after the introduction of the quality and outcomes framework: the alignment of financial incentives with professional values; concerns about changes to clinical practice; and the impact of surveillance within practices. Doctors and nurses generally reported that the quality and outcomes framework helped them provide what they regarded as high quality clinical care. Some concern was expressed that care might suffer from the introduction of targets that required respondents to do things that they did not regard as routine good clinical practice	Implementation of financial incentives for quality of care did not seem to damage the internal motivation of the general practitioners studied, although more concern was expressed by nurses	NR
McGregor et al., 2008 [124], UK, Qualitative	To investigate how practice nurses perceive the changes in their work since the General Medical Services (GMS) contract, including the Quality and Outcomes Framework (QOF) contract's inception	Individual interviews	Primary care	Nurses	Nurses were positive about the way in which their role has developed since the new contract but there were concerns about incentives, in particular financial reward for the amount of work they had carried out, and about the impact of QOF on the patient–nurse relationship. Roles and incentives were discussed in relation to two issues: professional development and professional status. Most practice nurses felt they had expanded their role and taken on new skills, particularly in chronic disease management and data recording, since the implementation of the new GMS contract. This view was consistent across practices, regardless of the level of QOF achievement or the socioeconomic profile of the practice population	The new GMS contract increased responsibility of practice nurses increased responsibility. However, discontent about how financial gains are distributed and negative impacts on core values may lead to detrimental long-term effects on motivation and morale	NR
Mayo-Bruinsma et al., 2013 [125], Canada, Quantitative	To determine whether models of primary care service delivery differ in their provision of family-centered care (FCC) and to identify practice characteristics associated with FCC	Patient and provider surveys based on the Primary Care Assessment Tool	Community health centres (CHCs), in which physicians receive a set annual salary; health service organizations (HSOs), in which payment is capitation based; and family health networks (FHNs), in which remuneration is principally capitation based	General practitioners, nurses and nurse practitioners	This study suggested that organizational characteristics, such as the number of clinical services offered, nurse practitioners, and family physicians, as well as the rural nature of the practice, can influence provider-reported FCC. Patient-reported FCC was not significantly different across primary care models and was mainly influenced by patient-level factors	Based on provider and patient reports, primary care reform strategies that encourage larger practices and more patients per family physician might compromise the provision of FCC, while strategies that encourage multidisciplinary practices and a range of services might increase FCC	To improve family-centered care healthcare leaders should promote multidisciplinary practices and the delivery of a range of services
Mohr et al., 2011 [126], USA, Quantitative	To test the hypothesis that aggregate job satisfaction of individuals comprising primary care teams is positively associated with quality of care, using a multilevel framework that nests patients within teams and examines both preventive measures of quality and biological markers	Secondary data analysis using the VA External Peer Review Program (EPRP) database for patient-level quality-of-care scores and the 2007 VA All Employee Survey (AES) for health care team member ratings of job satisfaction	Primary Care Teams	Physicians, mid-level providers, nurses, and support staff who have responsibility for a defined panel of patients	Aggregate team member satisfaction ratings were positively associated with higher scores for both process and intermediate outcome quality measures in a primary care setting. Team member satisfaction was found to be a robust predictor, as it was associated with both process and intermediate outcome quality measures. The parameter estimate for aggregate team member satisfaction was significant, albeit modest, when regressed on measures of quality. Community outpatient clinics were negatively associated with intermediate outcome quality measures compared to parent medical facilities	Team-level job satisfaction ratings are a potentially important marker for the effectiveness of primary care teams in managing patient care	Enhance job resources or job characteristics, e.g., providing more job-related training, financial rewards, or allowing more participation in decision-making processes Reduce job demands, e.g., allowing more time to complete job tasks, ensuring job roles and tasks are clear, or reducing workload by making changes or additions to staff
Mundt et al., 2015 [127], USA, Quantitative	To evaluate the associations between primary care team communication, interaction, and coordination (ie, social networks); quality of care; and costs for patients with cardiovascular disease	Sociometric survey	Primary care clinics	155 health professionals from 31 teams at 6 primary care clinics	Teams with higher density of daily interactions (face to face) among all team members and lower centralization were associated with better quality of care. Specifically, teams with more members reporting daily interactions with a greater number of team members show better quality of care, as measured by a 38% reduction in hospital days and $516 less spent on average per patient in the previous 12 months. Team shared vision about goals and commitments mediated the connection between team social network structures and patient outcomes. In other words, dense daily team interactions with all team members, notably, face-to-face connections, contributed to the development of shared team vision on the team’s objectives and expectations, which was linked to better quality of cardiovascular disease care. Results indicate that neither individual professional excellence nor electronic health records solutions alone could produce desired improvements in quality of care	Primary care teams that are more interconnected and less centralized and that have a shared team vision are better positioned to deliver high-quality cardiovascular disease care at a lower cost	Future studies may wish to explore these variables further
Mundt et al., 2016 [128], USA, Quantitative	To determine whether primary care team communication and team climate are associated with health outcomes, health care utilization, and associated costs for patients with diabetes	30-min face-to-face structured questionnaire administered by a trained research assistant	Primary care clinics	Physicians, physician assistants, or nurse practitioners	Primary care teams with a greater number of daily face-to-face communication ties among team members were associated with 52% (Rate Ratio = 0.48, 95% CI: 0.22, 0.94) fewer hospital days and US$1220 (95% CI: -US$2416, -US$24) lower health-care costs per team diabetes patient in the past 12 months. In contrast, for each additional registered nurse who reported frequent daily face-to-face communication about patient care with the primary care practitioner, team diabetes patients had less-controlled HbA1c (Odds Ratio = 0.83, 95% CI: 0.66, 0.99), increased hospital days (RR = 1.57, 95% CI: 1.10, 2.03), and higher healthcare costs (β = US$877, 95% CI: US$42, US$1713). Shared team vision, a measure of team climate, significantly mediated the relationship between team communication and patient outcomes	Primary care teams which relied on frequent daily face-to-face communication among more team members, and had a single nurse communicating patient care information to the primary care provider, had greater shared team vision, better patient outcomes, and lower medical costs for their diabetes patient panels	Support face-to-face discussions, multiple times per day
Naccarella 2009 [129], Australia, Qualitative	To explore the types and the qualities of GP work-related relationships	Interviews	Primary health care teams	General practitioners (GPs) practice nurses, practice managers and receptionists	Four main types of GP work-related relationships emerged: clinical problem solving, obtaining metaknowledge, obtaining legitimisation, and validation. Key qualities of GP work-related relationships included the nominated providers’ competence, accessibility, goodwill, honesty, consistency and communication styles	The study highlights the complex nature of GP work-related relationships that underpin the development of a primary health care system. The types and qualities of work-related relationships could inform the way professional development programs build the skills of GP and other healthcare providers to develop relationships within multidisciplinary team-based care approaches. The structure of GP working relationships and the context within which they are embedded are important considerations for policy reform aimed at influencing the healthcare system.	Policy emphasises should move from using structural reforms such as prescribed service delivery processes and financial incentives to encourage teamwork
Naccarella et al., 2013 [130], Australia, Qualitative	To propose a framework to assist policymakers, educators, researchers, managers and health professionals in supporting team-based models of primary care within the Australian health care system	Literature	Primary care	Healthcare teams	A review of incentives for primary health care team service provision recommended, on the basis of limited evaluative evidence, that a key priority was to develop teamwork-focused evaluative tools and indicator sets. The review also suggested that investment was required in reviewing existing (international and Australian) teamwork-related, evidence-based, evaluative inventories, tools and methods for use in the Australian setting, as well as in developing and piloting a set of process and summative teamwork-evaluation indicators (at patient, provider, organisational, and systems levels) for use in the Australian setting	Current Australian health care policy reforms continue to emphasise team-based primary care, and the proliferation of team-based models and investments designed to sustain their implementation require a robust framework of support. The framework proposed is an evidence-informed way to assist policymakers, educators, researchers, managers and health professionals to support team-based models of primary care within the Australian health care system. The framework is to be followed as a recipe, without reflection; rather, it is as a set of ingredients to support the implementation and sustainability of team-based models of primary care	NR
Oandasan et al., 2009 [131], Canada, Qualitative	To explore the impact of space and time on interprofessional teamwork in three primary health care centres and the implications for Canadian and other primary health care reform	Ethnographic observations; interviews	Academic family health centres	Three academic family health centres participated in a total of 139 hrs of observation and 37 interviews. Team members in all three centres from the disciplines of medicine, nursing, physiotherapy, occupational therapy, social work, dietetics, pharmacy, and office administration participated in this study	The study found that both the quantity and quality of interprofessional communication and collaboration in primary health care is significantly impacted by space and time. Across the three research sites, the physical layout of clinical space and the temporal organization of clinical practice led to different approaches to, and degrees of success with, interprofessional teamwork. Varied models of interprofessional collaboration resulted when these factors came together in different ways	The variability in team collaboration, which results from the interaction of temporal and spatial factors, has important implications for the transition of primary health care centres into Family Health Teams.ore likely to collaborate effectivel We found that providers in smaller interprofessional environments where providers are visible to one another and work from a reasonable proximity (not too far but not too close), are more interactive, both professionally and socially, and are more likely to collaborate effectively.	These findings have important implications for the transition to interprofessional family health teams in Canada and beyond
O’Brien et al., 2016 [132], Canada, Qualitative	To add to knowledge regarding what components make up a high functioning interprofessional primary care team	Literature Review; Interviews; Focus groups	Primary care	Physicians, nurses, allied health members, administrative and leadership personnel	A practice environment where members of an interprofessional teamwork in close proximity (co-location) was seen as enabling team high functioning. Perceived benefits to patients of co-location included the ability of providers to deal concurrently and comprehensively with patients’ needs; a reduction in the number of missed appointments and referrals; more timely provision of care and reduced duplication of services. Co-location was also seen as providing benefits to providers including facilitated communication and collaboration and the creation of informal professional development opportunities	While this study focused on physician-led teams it was intended to stimulate a wider conversation about what makes for good primary care and effective teaming in a variety of settings	The process of learning from these high-functioning primary care teams can inspire the efforts of others, encourage reflection and spark new conversations about how to navigate the team improvement journey from good to great
Pereira and Oliveira, 2018 [133], Portugal, Qualitative	To assess how Primary Health Care (PHC) nurses identify their professional autonomy in daily work and how this autonomy is perceived by other professionals of the multiprofessional team	Semi-structured interviews	Primary Health Care; Family Health Support Centres	27 nurses from the Family Health Strategy (FHS) and ten professionals from the Family Health Support Center	The findings revealed the professional autonomy of PHC nurses is perceived in the following categories: the possible autonomy, the autonomy dictated by protocols and the subordination to medical work	The study showed an expansion of the clinical scope of PHC nurses, and to a certain extent, it was closer to medical work. On the other hand, nurses are challenged to overcome such an approximation in the sense of interprofessional collaborative practice and advanced practice nursing	NR
Phipps-Taylor and Shortell, 2016 [134], USA, Qualitative	To explore the types of motivators that leaders use to stimulate change within accountable care organizations (ACOs)	Semi-structured interviews	ACOs	Physicians	The case study ACOs more strongly emphasized non-financial motivators for changing physician behavior than financial incentives. These motivators included mastery and social purpose, which were used frequently across all case study sites. Overall, the ACO case studies illustrated variability across all motivational domains. While there was evidence of changing motivators as a result of the ACO, the case study ACOs found it difficult to comprehensively change the use of motivators, in part due to dispersed managerial attention and the complexity and diversity of programs and contracts that fragmented efforts to improve	Motivating behavior change within ACOs goes beyond financial incentives. ACOs are using a broad range of motivators, including creating ways to make a greater impact on patients and opportunities to be a more effective physician. Overall, it does not appear that ACOs are deploying the full range of available motivators	Develop more sophisticated and wider‐ranging portfolios of motivators to drive behavior change
Pullon et al., 2008 [135], New Zealand, Qualitative	To investigate the roles of nurses and doctors, as well as the relationships between nurses and doctors, in New Zealand primary care settings	In-depth interviews	Primary Care Settings	Nurses and doctors in primary care settings	Three primary domains of extrinsic factors affected relationships between nurses and doctors: organizational and funding structures of the health system, organizational and employment issues at the practice level, and training and education issues. Trust was also a major theme discussed by participants, especially in relation to respect	Relationships between nurses and doctors that are marked by trust are established in a sequential way between individuals	Highly functional interprofessional relationships have the potential to become the reality with active support at the health system, educational, and professional organization level
Pullon et al., 2009 [20], New Zealand, Qualitative	To explore perceptions of interprofessional relationships, teamwork, and collaborative patient care in New Zealand primary care practice	In-depth interviews	Primary Care Practices	Individual nurses and doctors working in primary care settings	Nurses and doctors working in New Zealand primary care perceive funding models that include fee-for-service, task-based components as strongly discouraging collaborative patient care. In contrast, teamwork was seen to be promoted when health services, not individual practitioners, were bulk-funded for capitated healthcare provision. In well-organised practices, where priority was placed on uninterrupted time for meetings, open communication, and interprofessional respect, good teamwork was more often observed. Salaried practices, where doctors and nurses alike were employees, were considered by some interviewees to be particularly supportive of good teamwork	Health system, funding, and organisational factors act as significant barriers to the successful implementation of, and training for, effective teamwork in New Zealand primary care settings, despite new opportunities for more collaborative ways of working	More interprofessional education and professional development is needed to promulgate good business practice, and training in teamwork. It is essential that primary care nurses and doctors, as well as other primary healthcare professionals including pharmacists, physiotherapists, and midwives, are well-trained to work together, and well-supported to practice in effective teams
Pullon et al., 2016 [136], New Zealand, Qualitative	To determine how interprofessional collaboration (IPC) is achieved and maintained in general practices	Field notes, video-recordings, and transcripts	General Practices	General practice teams	Five overarching and intersecting cross-case themes emerged as key elements of IPC at practice level, with each having helpful and challenging aspects. Three themes concerned contextual and organisational factors, and two represented factors intrinsic to people within practices and/or teams	In an “all of practice” approach, opportunities for major changes in physical space design or employment models only arise occasionally, but much organisational change is achievable where staff have shared goals. Multiple opportunities for frequent, often brief, shared interprofessional communication should be facilitated by as many routes as possible. Direct observational methods hold promise in furthering knowledge and understanding of IPC in primary care practice, with potential to make explicit the connections between organisational, spatial, and temporal elements and their relationship to interpersonal/intrinsic factors	Attention needs to be paid to intrinsic individual and team characteristics, organizational, physical, and community environment in which the primary care practice functions
Rioux-Dubois and Perron, 2021 [137], Canada, Qualitative	To examine the integration and negotiation of the role of nurse practitioners in interprofessional primary healthcare settings	Semi-structured interviews (*n* = 23 nurse practitioners), direct observation, and document analysis	Community Health Centers (CHCs), Family Health Teams (FHTs), and NP-Led Clinics (PILCs)	Nurse practitioners, physicians	Organizational aims, practice standards, nurse practitioners’ right to self-determination, collaborative dynamics with physicians, and patient management were identified as integration factors that produced greater instability, needs for negotiation, and professional, identity, and moral difficulties for nurse practitioners	The results of this study challenge the commonly held belief that the role of nurse practitioners lacks clarity	Support flexible schedules, and role clarity for nurse practitioners
Rioux-Dubois and Perron., 2022 [138], Canada, Qualitative	To describe the enactment of interprofessional collaboration (IPC) in primary care settings, particularly as it relates to nurse practitioner (NP) integration	330 hrs of direct observation, 23 semi structured interviews, and document analysis	Community Health Centers (CHC), Family Health Teams (FHT), and Nurse Practitioner-Led Clinics (NPLC)	Physicians, nurses and nurse practitioners	The study shows that organizational care models with different mandates, strategic directions, remuneration models, and team sizes and composition, form parts of complex networks of human and non-human actors that give shape and meaning to IPC. Non-human actors, such as coffee machines and physical/virtual spaces, are active and powerful contributors to IPC. The study also shows that clinicians and managers could strengthen IPC by implementing administrative and clinical strategies that formalize IPC; defining IPC and its processes, and protecting time between collaborating partners	Organizational mandates and remuneration models, physical spaces and schedules played a decisive role in the enactment of IPC. Power structures embedded in certain designations (i.e., most responsible provider) or nurse practitioners commitments to physicians’ practices stood in contrast with the principles of IPC. Nurse Practitioners enacted various roles to develop, enhance, and maintain IPC. IPC remains poorly defined and precariously sustained	Both clinicians and managers should prioritize and carefully monitor the necessity and effective functioning of various forms of professional collaboration
Roland et al., 2006 [139], UK, Mixed Methods	To describe initial changes and predict the consequences of general practice models of care that may follow from the introduction of quality incentives	Telephone semi-structured interviews; survey questionnaires	General Practice Clinics	General Practitioners (GPs)	GPs believed the new contract will have a positive affect on their quality of care in targeted areas but an adverse affect on their professional autonomy and work-load. There was little variation in this view across GPs with different demographic or practice characteristics	GPs believe the new contract will have a positive affect on their quality of care in targeted areas	NR
Rosenthal et al., 2005 [140], USA, Quantitative	To evaluate the impact of a prototypical physician pay-for-performance program on quality of care	Reports analysis	Physicians	Physician groups	The findings give rise to a number of speculations about the effects of pay-for-performance. First, groups with baseline performance already above the targeted threshold understood that they needed only to maintain the status quo to receive the bonus payments. Low-performing groups improved as much as they did, given that their short-run chances of receiving the bonus were likely to be low. One possibility is that the groups viewed the quality improvement program as a larger signal of a changing environment in which they would face increasing pressure to improve their care systems and decided to begin moving in that direction. Paying explicitly for quality improvement might alter the incentives for high-performing and low-performing groups, distribute bonus dollars more toward the latter group, and possibly increase the overall impact of pay-for-performance	Paying clinicians to reach a common, fixed performance target may produce little gain in quality for the money spent and will largely reward those with higher performance at baseline	Continue experimentation with pay-for-performance
Russell et al., 2009 [141], Canada, Mixed Methods	(1) To assess whether chronic disease management differed among 4 models of primary health care delivery and (2) To identify which practice organizational factors were independently associated with high-quality care	Chart review, questionnaires, and semi-structured interviews	Community Health Centres (CHCs), Family Health Networks, Health Service Organizations	GPs and Nurse Practitioners	Chronic disease management was superior in CHCs. Clinicians in CHCs found it easier than those in the other models to promote high-quality care through longer consultations and interprofessional collaboration. Across the whole sample and independent of the model, high-quality chronic disease management was associated with the presence of a nurse-practitioner. It was also associated with lower patient-family physician ratios and when practices had 4 or fewer full-time-equivalent family physicians	The study supports the value of nurse-practitioners within primary care teams and validates the contributions of Ontario's CHCs. The observation that quality of care decreased in larger, busier practices suggests that moves toward larger practices and greater patient-physician ratios may have unanticipated negative effects on processes of care quality	Policy makers should support nurse-practitioners within primary care teams As moves toward larger practices and greater patient-physician ratios may have unanticipated negative effects, focusing on optimizing smaller clinics may be beneficial
Savageau et al., 2016 [142], USA, Quantitative	To identify factors related to preparedness, recruitment and retention	Online survey of 170-items was sent to Primary care providers	Community health centers (CHCs)	Family medicine, internal medicine, pediatrics, and obstetrics/ gynecology physicians	Beyond provider characteristics, several factors were important in retaining providers at CHCs. These factors included nonclinical interests in research and teaching, greater satisfaction with employee morale, the CHC model of care, recognition of clinical practice goals, professional development, and the availability of mentoring and feedback	The study suggests that CHCs should focus on factors such as mission, competency of peer physicians, teamwork, and supportive leadership in their recruitment and retention efforts. By addressing these factors, CHCs may be able to attract and retain providers who are committed to their mission and are satisfied with their work environment	Leaders should show interest in candidates who prioritize a shared mission and values Opportunities to learn from peers should be prioritized
Schadewaldt et al., 2016 [143], Australia, Mixed Methods	To investigate the experiences and perceptions of nurse practitioners and medical practitioners who worked together under the new policies and aimed to identify enablers of collaborative practice models	Direct observations, documents and semi-structured, questionnaires including validated scales	Collaborative practice models	Nurse practitioners, physicians, practice managers	Using the scale measurements, nurse practitioners and medical practitioners reported high levels of collaboration, were highly satisfied with their collaborative relationship and strongly believed that collaboration benefited the patient. The three themes developed from qualitative data showed a more complex and nuanced picture: 1) Structures such as government policy requirements and local infrastructure disadvantaged nurse practitioners financially and professionally in collaborative practice models; 2) Participants experienced the influence and consequences of individual role enactment through the co-existence of overlapping, complementary, traditional and emerging roles, which blurred perceptions of legal liability and reimbursement for shared patient care; 3) Nurse practitioners’ and medical practitioners’ adjustment to new routines and facilitating the collaborative work relied on the willingness and personal commitment of individuals	Findings of this study suggest that the willingness of practitioners and their individual relationships partially overcame the effect of system restrictions	Healthcare reform decision-makers should provide strategic support to enhance the roles of nurse practitioners and secure the long-term viability of collaborative practice models in primary healthcare
Shaw et al., 2005 [144], UK, Qualitative	To see whether primary healthcare professionals in these practices felt that progress with Personal Medical Services (PMS) was underpinned by effective teamworking	Semi-structured interviews	Personal Medical Services practices	Primary care professionals	Some participants felt they had used PMS to build their teams and develop quality based patient care. For other practices teamworking was limited by the absence of a common goal, recruitment difficulties, inadequate communication and hierarchical structures, and prevented practices from moving forward with clear direction	The study indicates that changing the contractual arrangements does not necessarily improve teamworking. It highlights the need for more sustained educational and quality improvement initiatives to encourage greater collaboration and understanding between healthcare professionals	NR
Shortell et al., 2004 [145], USA, Quantitative	To examine both the correlates of self-assessed or perceived team effectiveness and its consequences for actually making changes to improve care for people with chronic illness	Data analysis from program participation; Chronic Care Model (CCM)	Chronic Care Practices	Chronic Care team	A focus on patient satisfaction, the presence of a team champion, and the involvement of the physicians on the team were each consistently and positively associated with greater perceived team effectiveness. Maintaining a balance among cultural values of participation, achievement, openness to innovation, and adherence to rules and accountability also appeared to be important. Perceived team effectiveness, in turn, was consistently associated with both a greater number and depth of changes made to improve chronic illness care	The data suggest the importance of developing effective teams for improving the quality of care for patients with chronic illness	Research that examines patient physiological and patient satisfaction outcomes as a function of perceived team effectiveness and the number and types of changes actually made to improve care would further validate the importance of health care teams
Song et al., 2017 [146], USA, Mixed methods	To investigate the connections between team dynamics, job satisfaction of primary care providers (PCPs), and patient care coordination among PCPs in 18 primary care practices affiliated with Harvard that took part in Harvard's Academic Innovations Collaborative	Cross-sectional Survey and Qualitative Interviews	Primary Care Providers	Primary care physicians, nurse practitioners, and physician assistants, and resident physicians	There was a significant correlation between positive team dynamics and high job satisfaction among PCPs. Better patient care coordination was linked to higher levels of job satisfaction among PCPs. The study also revealed that patient care coordination mediated the relationship between team dynamics and job satisfaction of PCPs	To improve the overall functioning of a primary care team, it is essential to focus on enhancing work processes such as accountability, communication, information exchange, and conflict resolution. These are the areas with the lowest average level in terms of team dynamics. If a team is formed without proper planning, these crucial elements may be overlooked, leading to less than optimal team dynamics	It is important that healthcare leaders focus on improving primary care team dynamics
Taylor et al., 2015 [147], USA, Quantitative	To describe the implementation and impacts of Comprehensive Primary Care over its first year	Data feedback; the amount and format of feedback provided in other payers’ reports varied widely within regions	Comprehensive Primary Care (CPC) that involves 1) access and continuity, (2) planned chronic and preventive care, (3) risk-stratified care management, (4) patient and caregiver engagement, and (5) coordination of care across the medical neighborhood	Physicians, nurse practitioners, and physician assistants	This practice brought together 31 distinct payers (ranging from 3 to 9 per region) to collaborate in providing non-visit-based monthly care management fees, in addition to traditional payments, to support practices in their efforts to redesign and transform care. In the initial program year of CPC, this funding accounted for approximately 19 percent of total practice revenue (excluding CPC) or around $70,045 per clinician. Furthermore, CPC offers learning activities and data feedback on cost, service utilization, care quality, as well as patient, provider, and staff experiences to aid practices in their transformation journey. While there is room for improvement in learning activities and data feedback, ongoing refinements are being made. At the end of the first year, the majority of practices successfully achieved the required milestones, with fewer than 10 percent being placed on corrective action plans (38 practices) or terminated from the initiative (4 practices). Practice participation has remained stable, considering the substantial workload required to meet CPC's annual milestones. Payer participation has also shown consistency, with only a few payers discontinuing their involvement, and these payers had relatively small numbers of attributed patients in CPC	There was a notable but not statistically significant decrease (4 percent) in unplanned 30-day readmissions across the CPC program. However, there were limited significant effects observed on other quality-of-care outcomes or process measures assessed, which reflect the care provided by all healthcare providers involved in treating the patients	Policymakers should support learning about team-based care
Unützer et al., 2012 [148], USA, Quantitative	To evaluate a quality improvement program with a pay-for-performance (P4P) incentive in a population-focused, integrated care program for safety-net patients in 29 community health clinics	Quasi-experimental design, data analysis	Community health clinics	Patients	The analysis suggests that the institution of a quality improvement program with a P4P incentive substantially improved the quality and outcomes of care provided by the program. After the institution of the P4P incentive program, participants were substantially more likely to experience a significant improvement in depression severity, and the time to improvement was dramatically reduced compared with before the P4P incentive was implemented. These improvements in clinical outcomes were consistent with improvements observed in the quality of care that were the intended aims of the P4P initiative, such as early follow-up and psychiatric consultation for patients who were not improving	When clinical outcomes and key quality indicators are routinely tracked and a substantial portion of the payment for care is tied to quality indicators such as adequate follow-up and consultation for patients who are not improving, the quality and effectiveness of such programs can be substantially improved	NR
Valentijn et al., 2015 [149], Canada, Mixed methods	1) To develop a typology of integrated care projects (ICPs) based on the final degree of integration as perceived by multiple stakeholders. 2) To study how types of integration differ in changes of collaboration processes over time and final perceived effectiveness	Surveys and interviews	Integrated care projects	General practitioners, nurse, social worker and allied health professionals	ICPs within the United Integration Process subgroup made the strongest increase in trust-based (mutual gains and relationship dynamics) as well as control-based (organisational dynamics and process management) collaboration processes and had the highest overall effectiveness rates. ICPs with the Disunited Integration Process subgroup decreased on collaboration processes and had the lowest overall effectiveness rates. ICPs within the Professional-oriented Integration Process subgroup increased in control-based collaboration processes (organisational dynamics and process management) and had the highest effectiveness rates at the professional level	The research indicates that effective collaboration processes among stakeholders lead to shared perspectives and higher rates of effectiveness over time. On the other hand, when there are divergent perspectives at the professional, organizational, and system levels, trust-based and control-based collaboration processes can help align these perspectives. The study underscored the significance of acknowledging diverse viewpoints and employing various collaborative approaches when designing and implementing integrated care initiatives to achieve favorable outcomes	Healthcare leaders should focus on cultivating trust Future research should explore the need of relational trust- versus transactional control-based collaboration mechanisms
Beales et al., 2011 [150], Canada, Qualitative	To improve interprofessional collaboration on family health teams (FHTs) and other evolving healthcare teams, by examining the effect of professional culture on FHT collaboration	In-depth semi-structured focus groups	Academic teaching hospital	Medicine, nursing, and allied health professions at the Family Health Centre and Diabetes Education Centre in a large academic teaching hospital	Three main themes emerged: professional culture; FHT culture; and resources. Professional culture cannot be neatly separated from one’s personal, social or professional history, which ties in with opinions of accountability, power and hierarchy. Structure and processes of the FHT that encourage collaborative processes; clearly articulated scopes of practice, skills, authority; clarifications of roles and responsibilities; and opportunities to develop team relationships are necessary to diffuse the tension that exists between professional and FHT cultures	FHTs are multidisciplinary groups co-located but with a lack of meaningful structures and processes to support collaboration. There is heavy physician dominance and physicians seem to adhere to old hierarchical structures and beliefs, consistent with their professional culture	Health care providers need to build collaborative competencies (e.g. role clarity, effective communication) to move a group of interdisciplinary health care providers toward being a highly performing interprofessional team
Wilson et al., 2005 [151], Canada, Mixed methods	To assess Canadian family physicians/ general practitioners’ (FPs/GPs) interest and involvement in interdisciplinary collaborative practice	Focus groups and survey	Interdisciplinary collaborative practice	FP/GP, nurse practitioners, pharmacist, others	In focus groups, FPs/GPs identified seven categories of issues related to interdisciplinary collaborative practice: quality and capacity of care, quality of work life, affordability, availability/accessibility of other health professionals, team-building processes, responsibility/accountability, and system resources. Survey responses from 300 of 583 FPs/GPs in the region (51%) showed substantial interest in working with other health professionals, but strikingly less frequent current working relationships	The large gap between the interest and willingness of FPs/GPs to collaborate and their current involvement in teamwork must be addressed if collaborative practice is to increase in line with the goals of primary care reform in Canada	Policy makers should close the gap between the interest and willingness of FPs/GPs to collaborate and their current involvement in teamwork
Wranik et al., 2017 [48], Canada, Qualitative	To develop a framework for the conceptualization and analysis of financial arrangements in interdisciplinary primary care teams	(i) Interviews with 19 primary care decision makers representing 215 clinics in three Canadian provinces, (ii) A research roundtable with 14 primary care decision makers and/or researchers, and (iii) policy documents	Interdisciplinary Primary Care Teams (IDPC Teams)	Physicians, nurses and other non-physicians	Emergent implementation issues discussed by respondents include: (i) centrality of budget negotiations; (ii) approaches to patient rostering; (iii) unclear funding sources for space and equipment; and (iv) challenges with community engagement	The identification of optimal financial arrangements must be contextualized in terms of feasibility and the implementation environment. Financial hierarchy, both overt and covert, is considered a barrier to collaboration	Future research must explore the interplay between financial and non-financial incentives Team funding should not just be tied to physician activities
Wranik et al., 2018 [152], Canada, Qualitative	To characterize the implications that financial arrangements have on the balance of power in teams and whether financial models were perceived to influence the presence of professional hierarchies	(i) Policy documents describing financial/remuneration models in Interdisciplinary Primary Care (IDPC) teams across Canada, (ii) Semi-structured interviews	Primary care networks	Medical doctors, nurses, and other health care providers across three provinces	The study found that fee-for-service funding models were associated with medical dominance in interdisciplinary primary care teams, as the physicians in these models held decision-making power and controlled the distribution of resources. Salary-based funding models were found to facilitate more equitable decision-making and resource allocation within interdisciplinary primary care teams. These models allowed for increased collaboration and teamwork among healthcare providers	The fee-for-service model was associated with higher medical dominance, while salary-based models facilitated more equitable decision-making and resource allocation within teams	Policymakers should implement policies that minimize financial hierarchies and streamline funding sources
Xyrichis et al., 2008 [153], UK, Qualitative	To explore factors that inhibit or facilitate interprofessional teamworking in primary and community care settings	Comprehensive review of quan/qual studies	Primary and community care settings	Primary care teams	Two main themes emerged that had an impact on interprofessional teamworking: team structure and team processes. Within these two themes, six categories were identified: team premises; team size and composition; organisational support; team meetings; clear goals and objectives; and audit. The complex nature of interprofessional teamworking in primary care meant that despite teamwork being an efficient and productive way of achieving goals and results, several barriers exist that hinder its potential from becoming fully exploited; implications and recommendations for practice are discussed	These findings can inform development of current best clinical practice	Further research needs to be conducted into multidisciplinary teamworking at both the team and organisation level, to ensure that enhancement and maintenance of teamwork leads to an improved quality of healthcare provision

Twelve studies (*n* = 12/71, 17%) assessed the influence of provider remuneration models in team-based PC on patient, provider, team, and system outcomes. The details are summarized in Table 4. Remuneration for salaried and FFS payment models were targeted to the individual, while blended capitation models could target the team or individual.

**Table 4:** Impact of provider remuneration models on outcomes

Citation	Remuneration	Team Outcomes	Patient Outcomes	Provider Outcomes	System Outcomes
Beaulieu, M.-D., Haggerty, J., Tousignant, P., Barnsley, J., Hogg, W., Geneau, R.,... Bonin, L. (2013). Characteristics of primary care practices associated with high quality of care. *Cmaj*, 185(12), E590-E596 [89]	Salaried Models (CLSC) (Indiviudal)	NR (not reported)	Salaried models were strongly associated with quality of care compared to FFS or enhanced FFS models	NR	NR
Dimitrovová, K., Perelman, J., & Serrano-Alarcón, M. (2020). Effect of a national primary care reform on avoidable hospital admissions (2000–2015): A difference-in-difference analysis. *Social Science & Medicine, 252*, 112908 [98]	Capitation model P4P incentives (Individual)	NR	Reduction in rate of urinary tract infection	NR	The study found no significant impact on reducing hospitalization rates for ambulatory care-sensitive conditions
Glazier, R. H., Kopp, A., & Hutchison, B. G. (2016). Comparison of family health teams to other primary care models, 2004/05 to 2011/12: desLibris [106]	Salaried Models (Individual)	NR	Community Health Centres (CHCs) had higher inpatient admission and readmission rates than FFS and blended capitated team models	NR	Results were mixed regarding emergency department visits
Glazier, R. H., Rayner, J., & Zagorski, B. M. (2012). Comparison of primary care models in Ontario by demographics, case mix and emergency department use, 2008/09 to 2009/10: Institute for Clinical Evaluative Sciences [39]	Salaried (CHCs) (Individual) Fee for Service (Family Health Groups) (Individual) Blended reimbursement (large capitation component and partial Fee for Service; found in Family Health Networks (FHNs) and Family Health Organizations (FHOs), Family Health Teams (FHTs) (Individual and Team)	NR	NR	NR	CHCs: Lower than expected ED visits FHGs: Lower than expected ED visits FHNs: Higher than expected ED visits (urban and rural areas) FHOs: Higher than expected ED visits (urban areas), lower than expected ED visits (rural areas) FHTs: Higher than expected ED visits (urban and rural areas)
Greene, J., Hibbard, J. H., & Overton, V. (2014). A Case Study of a Team-Based, Quality-Focused Compensation Model for Primary Care Providers. Medical Care Research and Review, 71(3), 207–223. 10.1177/1077558713506749 [109]	Fee for Service (FFS) (Individual) Quality Based Compensation (Individual and Team)	Quality Based Compensation results in more collaboration with colleagues	FFS increases volume and reduces time with patients. Quality Based Compensation improves the quality of care	NR	NR
Khan, A. I., Barnsley, J., Harris, J. K., & Wodchis, W. P. (2022). Examining the extent and factors associated with interprofessional teamwork in primary care settings. *Journal of Interprofessional Care, 36*(1), 52–63. 10.1080/13561820.2021.1874896 [113]	Salaried model transitioning to a blended capitation model (Individual to team)	Higher collaboration is noted as it relates to similar purpose, goals; general relationships; team leadership (team process); general role responsibilities, autonomy (task feature); communication and information exchange (team process); community linkages and coordination of care (team process); decision-making and conflict management (team process); patient involvement) Team size, centralized administrative processes, high level of information exchange and mixed governance had a negative impact on collaboration	NR	NR	NR
Mayo-Bruinsma, L., Hogg, W., Taljaard, M., & Dahrouge, S. (2013). Family-centred care delivery: comparing models of primary care service delivery in Ontario. *Canadian Family Physician, 59*(11), 1202–1210 [125]	Salaried (Individual), FFS (Individual), blended capitation(Team)	NR	The salaried and team model resulted in higher mean provider-reported Family-Centered Care scores than all the other primary care models. Patient-reported FCC scores did not vary by model. NPs and on-site clinical services are associated with higher FCC scores	NR	NR
Pullon, S., McKinlay, E., & Dew, K. (2009). Primary health care in New Zealand: the impact of organisational factors on teamwork. *British Journal of General Practice, 59*(560), 191–197 [20]	Fee for Service (FFS) (Individual)	Nurses and doctors felt FFS discouraged collaboration	NR	NR	NR
Russell, G. M. M. F. M. F. M. P., Dahrouge, S. M., Hogg, W. M. M. M. D. F., Geneau, R. P., Muldoon, L. M. D. M. P. H. F., & Tuna, M. P. (2009). Managing Chronic Disease in Ontario Primary Care: The Impact of Organizational Factors. *Ann Fam Med, 7*(4), 309–318. 10.1370/afm.982 [141]	Salary (Individual) Fee for Service (Individual) Blended Capitation (Team)	NR	The salaried model shows better chronic disease management, explained by better performance on evidence-based processes associated with diabetes care; Greater perceived longer consultation with chronically ill patients	NR	NR
Schadewaldt, V., McInnes, E., Hiller, J. E., & Gardner, A. (2016). Experiences of nurse practitioners and medical practitioners working in collaborative practice models in primary healthcare in Australia–a multiple case study using mixed methods. *BMC family practice, 17*, 1–16 [143]	Salary models (Individual) Fee for Service (FFS) (Individual)	Salaried models enabled teamwork	NR	FFS does not compensate providers for collaborations with mutual patients	NR
Wranik, W. D., Haydt, S. M., Katz, A., Levy, A. R., Korchagina, M., Edwards, J. M., & Bower, I. (2017). Funding and remuneration of interdisciplinary primary care teams in Canada: a conceptual framework and application. *BMC health services research*, 17(1), 1–12 [48]	Fee for Service (Individual) Fee for Service with capitation payment (Individual and/or team) Salaried (Individual)	Financial Hierarchy	NR	NR	NR
Wranik, W. D., & Haydt, S. M. (2018). Funding models and medical dominance in interdisciplinary primary care teams: qualitative evidence from three Canadian provinces. *Human resources for health*, 16(1), 1–9 [152]	Fee for Service (FFS) (Individual) Fee for Service with capitation payment (Individual and/or team) Salaried (Individual)	Salaried models enable interprofessional relationships	NR	FFS results in less delegation of activities to team members	NR

Several qualitative studies indicate salaried models are more conducive to teamwork [48, 143, 152]. These studies emphasized how non-hierarchical payment and funding models facilitated collaboration between physicians and nurses [48] and reduced financial hierarchy [152]. Studies show that FFS remuneration increases volume [109], discourages referrals to non-physician providers [48] and team collaboration [20] and does not sufficiently compensate for shared patients [143]. A study that examined the impact of shifting from FFS to a quality-based compensation model (payment for performance) [109] found the model was perceived to improve collaboration with colleagues and increase quality care but lowered the satisfaction of physicians [109]. A study from Portugal found the shift from salaried remuneration to blended capitation did not significantly affect avoidable hospitalization rates for ambulatory care-sensitive conditions [98].

In Canada, several studies evaluated team models with different provider remuneration models with respect to team, provider, patient, and system outcomes. However, the level of maturity, self-selection into models and confounding of remuneration and organizational models make interpretation difficult. It is important to note that these studies did not explicitly establish causal inferences regarding the impact of these remuneration models on outcomes. A quantitative study assessing team collaboration in different PC models in Ontario, Canada, revealed that Community Health Centers (CHC; a salaried model) scored significantly higher on the Collaborative Practice Assessment Tool (CPAT) compared to Family Health Teams (FHTs) (blended capitation) [113]. These findings were attributed to the maturity of the models, with the FHT model being less mature in developing inter-dependency between professionals, affecting teamwork.

A survey of providers found that CHCs outperformed other models in terms of provider-reported Family-Centered Care (FCC) scores [125]. However, patient-reported family-centered care (FCC) scores exhibited no significant differences [125]. CHCs' performance was attributed to clinical services, after-hours access, and nurse practitioners [125]. On system outcomes, the salaried CHC model, compared to blended capitation and enhanced FFS models, served a population with a higher proportion of disadvantaged and sicker individuals, recent immigrants, and patients with co-morbidities [39] and fewer emergency department visit rates [39]. CHCs also had significantly higher community orientation scores compared to FFS and blended capitation models [154]. However, another study found that CHCs had higher inpatient admission and readmission rates compared to FHTs [106]. The outcomes could have been influenced by a variety of factors, including residual confounding, health-promoting services, community engagement, and the nature of appointment scheduling [106].

### Extrinsic incentives and impact on outcomes

**Fig. 2:**
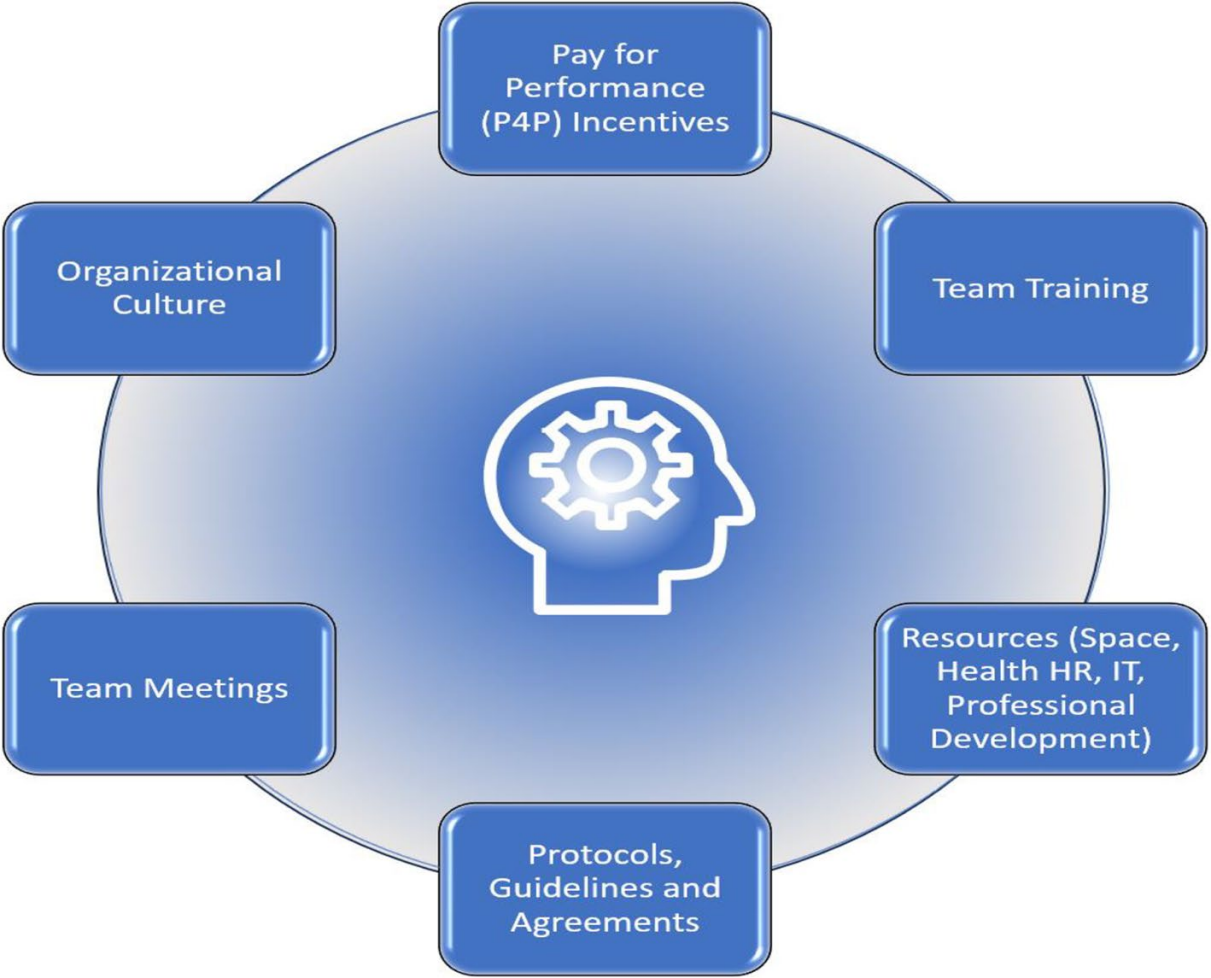
Extrinsic incentives and impact on outcomes

Fifty-four (*n* = 54/71,76%) articles identified extrinsic incentives in PC teams and their impact on outcomes. The details are summarized in Table 5 and Fig. 2.

**Table 5:** Extrinsic incentives in interprofessional primary care teams

Citation	Extrinsic Incentives (Level of Incentive, if specified or inferred)	Team Outcomes	Patient Outcomes	Provider Outcomes	System Outcomes
Arevian, M. (2005). The significance of a collaborative practice model in delivering care to chronically ill patients: a case study of managing diabetes mellitus in a primary health care center. *Journal of interprofessional care, 19*(5), 444–451 [87]	Team Meetings (Team)	Team meetings provide an opportunity for shared decision-making and the development of respect	Not Reported (NR)	NR	NR
Bareil, C., Duhamel, F., Lalonde, L., Goudreau, J., Hudon, E., Lussier, M.-T.,... Lalonde, G. (2015). Facilitating implementation of interprofessional collaborative practices into primary care: A trilogy of driving forces. *Journal of Healthcare Management, 60*(4), 287–300. [88]	Organized and Facilitated Team-Meetings (Team)	Positive: Increased collaboration (team process) between general practitioners and nurses Fostered team dialogue (team process), establishing roles and responsibilities	Mixed: 11% decrease in primary care visits and 6% decrease in specialist visits for family medicine group enrollees. No evidence of an effect on hospitalizations or associated costs	Not Reported (NR)	Mixed: Supports the idea that primary care organizational reforms can impact the healthcare system without changing physician payment mechanisms; however, no evidence of overall healthcare cost savings No evidence of an effect associated with costs
Campbell, S. M., McDonald, R., & Lester, H. (2008). The experience of pay for performance in English family practice: a qualitative study. *The Annals of Family Medicine*, 6(3), 228–234 [91]	Financial Incentives (Individual)	Resentment by team members not benefiting financially from payments	NR	Changed behaviour for nurses and general practitioners, including improvements in disease-specific processes of patient care and physician income Potential deskilling of doctors due to an enhanced role for nurses in managing long-term conditions	NR
Campbell, S. M., Kontopantelis, E., Reeves, D., Valderas, J. M., Gaehl, E., Small, N., & Roland, M. O. (2010). Changes in patient experiences of primary care during health service reforms in England between 2003 and 2007. *The Annals of Family Medicine*, 8(6), 499–506 [92]	Financial Incentives (Team)	NR	Patient sociodemographic characteristics (age), and practice-specific factors (practice size) impact performance	NR	NR
Campbell, S., Hannon, K., & Lester, H. (2011). Exception reporting in the Quality and Outcomes Framework: views of practice staff—a qualitative study. *Br J Gen Pract*, 61(585), 183–189. 10.3399/bjgp11X567117 [93]	Financial Incentives (Team)	NR	NR	Providers may exclude certain patients near the end of the payment year to meet remaining targets and prevent financial penalties	NR
Cashman, S. B., Reidy, P., Cody, K., & Lemay, C. A. (2004). Developing and measuring progress toward collaborative, integrated, interdisciplinary health care teams. *Journal of interprofessional care*, 18(2), 183–196 [94]	Team Training (Team) Team Meetings (Team)	Team meetings with an external facilitator improve collaboration. Team training coupled with dedicated team meetings resulted in high perceived team functioning across dimensions such as dominant vs. submissive, friendly vs. unfriendly, and acceptance vs. non-acceptance of task orientation of established authority	NR	NR	NR
Cassou, M., Mousques, J., & Franc, C. (2020) [95] General practitioners’ income and activity: the impact of multi-professional group practice in France.* The European Journal of Health Economics* 2020, 21:1295-1315.	Financial Incentives (Individual) Team Meetings (Team) Access to resources (Team)	Interprofessional facilitation. Team meetings allowed productive teamwork, enabling clinicians to get to know one another professionally and personally, stimulating team building. Facilitators played a crucial role in running efficient meetings and gathering information	Team meetings and patient access to additional professional services (nutritionists, kinesiologists, psychologists)	Physician motivation is increased	Potential cost-effectiveness of supported facilitation; interprofessional facilitation team worked as an implementation task force, allowing for small-scale interventions, testing them inside and outside the clinic, and improving the clinic's organizational change
Delva, D., Jamieson, M., & Lemieux, M. (2008). Team effectiveness in academic primary health care teams. *Journal of interprofessional care*, 22(6), 598–611 [96]	Team Meetings (Team) Protocols (Team)	Role clarity through documentation Team meetings improved efficiency	NR	NR	NR
Dieleman, S. L., Farris, K. B., Feeny, D., Johnson, J. A., Tsuyuki, R. T., & Brilliant, S. (2004). Primary health care teams: team members' perceptions of the collaborative process. *Journal of Interprofessional Care*, 18(1), 75–78 [97]	Organizational Culture (Team)	Effective collaboration and communication	NR	NR	NR
Doran, T., Campbell, S., Fullwood, C., Kontopantelis, E., & Roland, M. (2010). Performance of small general practices under the UK's Quality and Outcomes Framework. *British Journal of General Practice*, 60(578), e335-e344 [101]	Financial Incentives (Individual)	Patient sociodemographic characteristics, specifically age and practice-specific factors, such as practice size, impacted team performance	NR	NR	NR
Doran, T., Fullwood, C., Gravelle, H., Reeves, D., Kontopantelis, E., Hiroeh, U., & Roland, M. (2006). Pay-for-Performance Programs in Family Practices in the United Kingdom. *New England Journal of Medicine*, 355(4), 375–384. 10.1056/NEJMsa055505 [99]	Financial Incentives (Individual)	A few practices achieved high scores in pay for perfomance (P4P) by excluding a significant number of patients through exception reporting	The patient-to-practitioner ratio significantly impacted performance	High levels of achievement were attained in the first year of the P4P contract	NR
Doran, T., Fullwood, C., Kontopantelis, E., & Reeves, D. (2008) [100] Effect of financial incentives on inequalities in the delivery of primary clinical care in England: analysis of clinical activity indicators for the quality and outcomes framework. *The Lancet, *372(9640):728-736.	Financial Incentive (Individual)	Improved timework	No clinically significant differences in preventive care quality between incentivized and no incentivized clinics. Most physicians felt the incentives were ineffective in improving the quality of care		No evidence of a clinically significant effect of financial incentives on the performance of preventive care
Drew, P., Jones, B., & Norton, D. (2010). Team effectiveness in primary care networks in Alberta. *Healthcare quarterly (Toronto, Ont.)*, 13(3), 33–38 [102]	Organizational Culture (Team) Team Meetings (Team)	Greater interprofessional collaboration	NR	NR	NR
Drummond, N., Abbott, K., Williamson, T., & Somji, B. (2012) [103] B: Interprofessional primary care in academic family medicine clinics: implications for education and training. *Canadian Family Physician*, 58(8):e450-e458.	Organizational Culture (Team) Resources (Team) Protocols, Guidelines and Agreements (Team)	Shared goals and vision, sense of belonging, governance, effective communication (team process), shared decision-making (team process), co-location, strong leadership (team process), and team meetings contribute to improved team impact	NR	NR	NR
Gemmell, I., Campbell, S., Hann, M., & Sibbald, B. (2009). Assessing workload in general practice in England before and after the introduction of the pay-for-performance contract. *Journal of Advanced Nursing*, 65(3), 509–515. 10.1111/j.1365-2648.2008.04902.x [104]	Financial Incentives (team)	Some nurses felt that the incentives improved teamwork	NR	Many nurses reported that the incentives did not increase their salaries. The incentives increased workloads for nurses, with higher visit rates but no change in the number of hours worked per week	NR
Gené-Badia, J., Escaramis-Babiano, G., Sans-Corrales, M., Sampietro-Colom, L., Aguado-Menguy, F., Cabezas-Pena, C., & de Puelles, P. G. (2007). Impact of economic incentives on quality of professional life and on end-user satisfaction in primary care. *Health Policy*, 80(1), 2–10 [105]	Financial Incentives (individual)	NR	NR	Physicians' perception of the burden of demands increased, leading to decreased satisfaction. Incentives for long-term professional development increased nurses' perception of support	NR
Goldman, J., Meuser, J., Rogers, J., Lawrie, L., & Reeves, S. (2010) [107] Interprofessionalcollaboration in family health teams: An Ontario-based study. *Canadian Family Physician*, 56(10).	Supportive management and leadership (Team). The physical layout and allocation of space (Team) Interprofessional initiatives (e.g., team policies, hiring processes, interprofessional education activities) (Team)	Rethinking traditional roles and scopes of practice leading to better communication (team process) and cooperation (team process) among team members. Improved collaboration (team process) due to strong management and leadership (team process); Better teamwork facilitated by appropriate time and space considerations; Enhanced collaboration (team process) due to interprofessional initiatives	NR	NR	NR
Grant, S., Huby, G., Watkins, F., Checkland, K., McDonald, R., Davies, H., & Guthrie, B. (2009). The impact of pay‐for‐performance on professional boundaries in UK general practice: an ethnographic study. *Sociology of Health & Illness*, 31(2), 229–245 [108]	Financial Incentives	NR	NR	English family practices have increased the employment of managerial roles	NR
Greene, J., Hibbard, J. H., & Overton, V. (2014) [109] A Case Study of a Team-Based, Quality-Focused Compensation Model for Primary Care Providers. *Medical Care Research and Review,* 71(3), 207-223. 10.1177/1077558713506749	Financial Incentives (individual)	Encouraged collaboration (team process) and teamwork among providers	Providers focus on preventive care and chronic disease management, resulting in better patient health outcomes	NR	Providers focus on preventive care and chronic disease management, resulting in reduced hospitalizations and fewer emergency room visits The new compensation model results in lower fee for service billing, potentially reducing healthcare costs
Hämel, K., & Vössing, C. (2017). The collaboration of general practitioners and nurses in primary care: a comparative analysis of concepts and practices in Slovenia and Spain. *Prim Health Care Res Dev*, 18(5), 492–506 [110]	Financial Incentives (Individual) Clear definitions of tasks and responsibilities, well-structured procedures, active and continuous communication (Team)	Teamwork, coordination (team process), and cooperation (team process) are supported organizational properties	Increased number of patients seen, resulting in improved access to primary care	General practitioners see and follow more patients without increasing the quantity of delivered services	Multi-professional team-based primary care addresses the shortage of medical time, particularly in underserved areas
Harris, M. F., Advocat, J., Crabtree, B. F., Levesque, J. F., Miller, W. L., Gunn, J. M.,... Russell, G. M. (2016). Interprofessional teamwork innovations for primary health care practices and practitioners: evidence from a comparison of reform in three countries. *J Multidiscip Healthc*, 9, 35–46. 10.2147/JMDH.S97371 [111]	Resources (Team) Team Meetings (Team)	Stronger relationships among team members. Enhanced proximity and communication within the team	NR	Meetings are perceived to support clinical and professional needs	NR
Hepp, S., Misfeldt, R., Lait, J., Armitage, G. D., & Suter, E. (2014) [112] Organizational factors influencing inter-professional team functioning in primary care networks. *Healthcare Quarterly* (Toronto, Ont), 17(2):57-61.	Physical infrastructure, information technology infrastructure, organizational supports (Team)	Co-location of team members in a Primary Care Network office facilitated collaboration, communication, and relationship building. Decentralization allowed relationships and trust to build between physicians and team members working together	NR	Positive impact due to leadership and workplace culture fostering encouragement, trust, and continuous quality improvement	NR
Kiran, T., Victor, J. C., Kopp, A., Shah, B. R., & Glazier, R. H. (2012). The relationship between financial incentives and quality of diabetes care in Ontario, Canada. *Diabetes care*. 2012 May 1;35(5):1038–46 [115]	Financial Incentives (Team)	Team-based care improves care processes overall	Moderate improvements in diabetes care. Effects on cancer screening are less clear, but team-based care improves care processes overall	NR	Positive impact on system outcomes due to improved care processes
Kirschner, K., Braspenning, J., Akkermans, R. P., Jacobs, J. A., & Grol, R. (2013). Assessment of a pay-for-performance program in primary care designed by target users. *Fam Pract*, 30(2), 161–171 [116]	Financial Incentives (Individual)	NR	In the Netherlands, an evaluation of general practices with indicators for chronic care, prevention, practice management, and patient experience found significant improvements in process indicators for cardiovascular risk management and asthma No significant improvements in the influenza vaccination rate and cervical cancer screening	NR	NR
LaMothe, J., Hendricks, S., Halstead, J., Taylor, J., Lee, E., Pike, C., & Ofner, S. (2021) [117] Developing interprofessional collaborative practice competencies in rural primary health care teams.* Nursing Outlook*, 69(3), 447-457.	Meetings (in-person) (Team) Evaluations (Team and Individual) Training and Education (Team)	Effective communication within a team encourages interprofessional practices and contributes to an improved team dynamic, reducing staff turnover. Collaboration is facilitated through practices like huddles, aligning goals, and fostering academic-practice partnerships	NR	Tight patient appointment schedules present barriers; competing demands limit readiness for changes	Leveraging resources of academic-practice partnership is key to project success
Lanham, H. J., McDaniel, R. R., Jr., Crabtree, B. F., Miller, W. L., Stange, K. C., Tallia, A. F., & Nutting, P. (2009). How improving practice relationships among clinicians and nonclinicians can improve quality in primary care. *Jt Comm J Qual Patient Saf*, 35(9), 457–466. https://doi.org/10.1016/ [118]	Organizational Culture (Team)	Team effectiveness relies on establishing a culture that fosters trust, mutual respect, and collaborative decision-making	NR	NR	NR
Lehtovuori, T., Kauppila, T., Kallio, J., Raina, M., Suominen, L., & Heikkinen, A. M. (2015) [119] Financial team incentives improved recording of diagnoses in primary care: a quasi-experimental longitudinal follow-up study with controls. *BMC research notes* 2015, 8(1):1-6.	Financial Incentives (Individual) Well-structured procedures (Team) Regular meetings (Team)	Positive: Clear task assignment**s** and well-structured procedures assisted in implementing new methods. Active and continuous communication (team process) prevented misunderstandings and backslides. Regular team meetings and the exchange of experiences helped strengthen teamwork	Patients experience improved quality of care	NR	The proportion of doctor visits with recorded diagnoses in the team increased from 55 to 90%
Lester, H., Matharu, T., Mohammed, M. A., Lester, D., & Foskett-Tharby, R. (2013). Implementation of pay for performance in primary care: a qualitative study 8 years after introduction. *British Journal of General Practice*, 63(611), e408-e415 [120]	Financial Incentives (Individual)	NR	NR	Some physicians prioritized their pay over patients' best interests. Reduced clinical autonomy due to increased micromanagement of the clinical workload	NR
Maisey, S., Steel, N., Marsh, R., Gillam, S., Fleetcroft, R., & Howe, A. (2008). Effects of payment for performance in primary care: qualitative interview study. *J Health Serv Res Policy*, 13(3), 133–139 [122]	Financial Incentives (Individual)	Substantial improvements in teamwork, consistency, and recording of care for incentivized conditions	Limited engagement with results of patient surveys or patient involvement initiatives. Limited improvements were observed for non-incentivized conditions Focus on record-specific clinical activities perceived to shift attention away from patients' concerns	Some participants described manipulating data to maximize practice income. Nurses experienced increased workload**s** but reported more autonomy and job satisfaction. Doctors acknowledged improved disease management but expressed unease about 'box-ticking' and increased demands of team supervision	NR
McDonald, R., Harrison, S., Checkland, K., Campbell, S. M., & Roland, M. (2007). Impact of financial incentives on clinical autonomy and internal motivation in primary care: ethnographic study. *Bmj*, 334(7608), 1357 [52]	Financial Incentives (Individual)	NR	NR	Did not affect the intrinsic motivation of physicians. Nurse Practitioners expressed more concerns regarding the changes to their clinical practice	NR
McGregor, W., Jabareen, H., O'Donnell, C. A., Mercer, S. W., & Watt, G. C. (2008). Impact of the 2004 GMS contract on practice nurses: a qualitative study. *Br J Gen Pract*, 58(555), 711–719. 10.3399/bjgp08X342183 [124]	Financial Incentives (Individual)	Improved teamwork	NR	Financial incentives expanded nurses' skills, particularly in chronic disease management and data recording. Nurses perceived increased status, with more autonomy and independence in their role. Nurses felt their role became more central in the practice. Many nurses reported that the incentives did not increase their salaries. The incentives resulted in increased workloads, with higher visit rates and no change in the number of hours worked per week	NR
Mundt, M. P., Agneessens, F., Tuan, W.-J., Zakletskaia, L. I., Kamnetz, S. A., & Gilchrist, V. J. (2016). Primary care team communication networks, team climate, quality of care, and medical costs for patients with diabetes: a cross-sectional study. *International Journal of Nursing Studies*, 58, 1–11 [128]	Shared Purpose (Team)	Improved team collaboration or team effectiveness. Shared team vision, a measure of team climate, mediated the relationship between team communication and patient outcomes	NR	NR	NR
Mundt, M. P., Gilchrist, V. J., Fleming, M. F., Zakletskaia, L. I., Tuan, W.-J., & Beasley, J. W. (2015). Effects of primary care team social networks on quality of care and costs for patients with cardiovascular disease. *The Annals of Family Medicine*, 13(2), 139–148 [127]	Organizational Culture (Team)	Dense daily team interactions, particularly face-to-face connections, contributed to developing a shared team vision of objectives and expectations	The development of a shared team vision was associated with better quality of cardiovascular disease care	NR	NR
Naccarella, L., Greenstock, L. N., & Brooks, P. M. (2013). A framework to support team-based models of primary care within the Australian health care system. *The Medical Journal of Australia*, 199(5), S22-S25 [130]	Resources (Team) Training (Team)	General practitioners rely on these work-related relationships to generate solutions to clinical problems (team psycho-social trait), obtain meta-knowledge, validate clinical decisions, and legitimize their actions when dealing with complex or chronic conditions	NR	NR	NR
O’Brien, P., Aggarwal, M., Rozmovits, L., Whittaker, M.-K., & Ellison, P. (2016). The teaming project: Learning from high-functioning interprofessional primary care teams. Retrieved from: https://dfcm.utoronto.ca/sites/default/files/The%20Teaming%20Project%20Report%202016-10-17.pdf [132]	Resources (Team) Team Meetings (Team) Professional Development (Individual)	Professional development was recognized as crucial for organizational functioning, Effective use of electronic medical records enhanced team functioning, efficiency, communication, continuity of care, and quality improvement initiatives	NR	NR	Managers acknowledged the associated costs of diverting team members from regular tasks
Oandasan, I. F., Conn, L. G., Lingard, L., Karim, A., Jakubovicz, D., Whitehead, C.,... Reeves, S. (2009). The impact of space and time on interprofessional teamwork in Canadian primary health care settings: implications for health care reform. *Prim Health Care Res Dev*, 10(2), 151–162 [131]	Resources (Team)	Facilitates collaboration	NR	NR	NR
Pullon, S. (2008). Competence, respect and trust: Key features of successful interprofessional nurse-doctor relationships. *Journal of Interprofessional Care, 22*(2), 133–147. 10.1080/13561820701795069 [135]	Professional Competence (Individual)	Trust was regarded as a factor that developed within the context of understanding and respecting professional competence	NR	NR	NR
Pullon, S., McKinlay, E., & Dew, K. (2009). Primary health care in New Zealand: the impact of organisational factors on teamwork. *British Journal of General Practice*, 59(560), 191–197 [20]	Team Meetings (Team)	Facilitates team functioning	NR	NR	NR
Pullon, S., Morgan, S., Macdonald, L., McKinlay, E., & Gray, B. (2016). Observation of interprofessional collaboration in primary care practice: a multiple case study. *Journal of interprofessional care*, 30(6), 787–794 [136]	Team Meetings (Team) Organizational Culture (Team)	Facilitates team functioning Interprofessional collaborative practice	NR	NR	NR
Rioux-Dubois, A., & Perron, A. (2021). The integration of nurse practitioners into primary health care: Rethinking the negotiation of complex dynamics. *Recherche en soins infirmiers*, 145(2), 38–52 [137]	Team Meetings (Team)	Facilitates team functioning	NR	NR	NR
Rioux-Dubois, A., & Perron, A. (2022). Enacting primary healthcare interprofessional collaboration: a multisite ethnography of nurse practitioner integration in Ontario, Canada. *Journal of interprofessional care*, 1–9 [138]	Resources(Team) Team Meetings (Team)	Facilitating and maintaining team functioning	NR	NR	NR
Roland, M., Campbell, S., Bailey, N., Whalley, D., & Sibbald, B. (2006). Financial incentives to improve the quality of primary care in the UK: predicting the consequences of change. *Prim Health Care Res Dev*, 7(1), 18–26 [139]	Financial Incentives (Individual)	NR	NR	Increased the employment of nurses and data entry clerks	NR
Rosenthal, M. B., Frank, R. G., Li, Z., & Epstein, A. M. (2005). Early Experience With Pay-for-PerformanceFrom Concept to Practice. *JAMA*, 294(14), 1788–1793. 10.1001/jama.294.14.1788 [140]	Financial Incentives (Individual)	NR	Enhanced clinical quality scores in the areas of cervical cancer screening and mammography	NR	NR
Russell, G. M. M. F. M. F. M. P., Dahrouge, S. M., Hogg, W. M. M. M. D. F., Geneau, R. P., Muldoon, L. M. D. M. P. H. F., & Tuna, M. P. (2009). Managing Chronic Disease in Ontario Primary Care: The Impact of Organizational Factors. *Ann Fam Med*, 7(4), 309–318. 10.1370/afm.982 [141]	Access to resources (Team)	Improved team functioning	NR	NR	NR
Savageau, J. A., Cragin, L., Ferguson, W. J., Sefton, L., & Pernice, J. (2016). Recruitment and retention of community health center primary care physicians post MA Health Care Reform: 2008 vs. 2013 physician surveys. *Journal of health care for the poor and underserved*, 27(3), 1011–1032 [142]	Financial Incentives (Indivdiual) Supportive leadership (Team) Work/life balance (Individual) Resources (Team) Opportunities for professional development (Team)	Enhanced teamwork and collaboration (team process); a smaller percentage of responders felt prepared to work in fully integrated teams due to the complexity of transformation efforts	NR	Improved retention, staff morale, and satisfaction due to work/life balance, support staff, operational support, information technology infrastructure, and data analytics	NR
Schadewaldt, V., McInnes, E., Hiller, J. E., & Gardner, A. (2016). Experiences of nurse practitioners and medical practitioners working in collaborative practice models in primary healthcare in Australia–a multiple case study using mixed methods. *BMC family practice*, 17, 1–16 [143]	Policies (Team)	High perceived collaboration (team process), high satisfaction with other health professionals, but less frequent current working collaborative relationships. A large gap between the interest and willingness of general practitioners to collaborate and their current involvement in teamwork must be addressed to increase collaborative practice in line with primary care reform goals	Potential for improved quality and capacity of care, more patient-centered approach to needs	Improved satisfaction with professional work, and the potential for better communication between professionals	Government policy requirement and local infrastructure; legal liability and reimbursement for shared patient care; role clarity
Shaw, A., De Lusignan, S., & Rowlands, G. (2005). Do primary care professionals work as a team: a qualitative study. *Journal of interprofessional care*, 19(4), 396–405 [144]	Team Culture (Team)	ierarchy impeded team working	NR	Reduced feelings of shared ownership among staff members	NR
Shortell, S. M., Marsteller, J. A., Lin, M., Pearson, M. L., Wu, S.-Y., Mendel, P.,... Rosen, M. (2004). The Role of Perceived Team Effectiveness in Improving Chronic Illness Care. *Medical Care*, 42(11), 1040–1048 [145]	Presence of Champions (Team) Team Culture (Team)	Improved efficiency	NR	NR	NR
Taylor, E. F., Dale, S., Peikes, D., Brown, R., Ghosh, A., Crosson, J.,... Shapiro, R. (2015). Evaluation of the Comprehensive Primary Care Initiative: first annual report. *Mathematica Policy Research* [147]	Trust (Individudal)	Facilitation of shared decision-making and coordinated actions	NR	NR	NR
Unützer, J., Chan, Y.-F., Hafer, E., Knaster, J., Shields, A., Powers, D., & Veith, R. C. (2012). Quality improvement with pay-for-performance incentives in integrated behavioral health care. *American journal of public health*, 102(6), e41-e45 [148]	Financial Incentive (Individudal and Team)	NR	Higher likelihood of patients receiving timely follow-up care. Time to achieve depression improvement was significantly reduced	NR	NR
Valentijn, P. P., Ruwaard, D., Vrijhoef, H. J., de Bont, A., Arends, R. Y., & Bruijnzeels, M. A. (2015). Collaboration processes and perceived effectiveness of integrated care projects in primary care: a longitudinal mixed-methods study. *BMC health services research, 15*, 1–12 [149]	Trust (Individual)	Higher effectiveness rates	NR	NR	NR
Beales J, Walji R, Papoushek C, Austin Z. Exploring professional culture in the context of family health team interprofessional collaboration. Health Interprofessional Pract Educ. 2011;1(1). [150]	Documents (Team) Team Culture (Team)	Improved processes	NR	Adherence to older beliefs	NR
Wilson, D. R., Moores, D. G., Lyons, S. C. W., Cave, A. J., & Donoff, M. G. (2005). Family physicians’ interest and involvement in interdisciplinary collaborative practice in Alberta, Canada. *Prim Health Care Res Dev*, 6(3), 224–231 [151]	Formal training workshops (Team). Increased meeting time(Team). Affordability, availability/accessibility of other health professionals (Team). Responsibility/accountability and system resources (Individual and Team).	Some improvements in friendliness, task orientation, decision-making (team process), and teamwork, but limited or negative progress in authority acceptance, loyalty, and self-interest	NR	NR	NR
Xyrichis, A., & Lowton, K. (2008). What fosters or prevents interprofessional teamworking in primary and community care? A literature review. *International Journal of Nursing Studies*, 45(1), 140–153 [153]	Organizational Culture (Team) Team Meetings (Team)	Improved teamwork	NR	NR	NR

### Pay-for-performance incentives

The majority of studies on P4P programs implied that incentives were offered at the individual level. P4P incentives at the individual level facilitated greater employment of nurses and data entry clerks, implementation of information technology [139] and managerial roles [108]. P4P incentives expanded the skills of nurses in chronic disease management and data recording, increased their perceived autonomy [124], improved job satisfaction [105, 124] and fostered teamwork [119, 122]. However, there were unintended consequences with the use of P4P incentives for providers and communities. P4P incentives increased the workload of nurses [104] [124] and unintentionally reduced their satisfaction [52]. It also potentially resulted in the de-skilling of GPs who delegated more chronic disease management activities to nurses [91]. Another unintended consequence was that the structure of incentives did not consider local context, which resulted in the inequitable distribution of incentives, with practices in more deprived populations receiving lower financial rewards [100]. Finally, the incentives resulted in the gaming of the system, with some practices achieving high scores by excluding patients from targets to achieve goals [93] for financial gain [122].

P4P incentives showed mixed results on the impact on clinical process indicators, with some studies showing some improvement in cardiovascular risk management, asthma [116] and diabetes [115]. In contrast, others showed no or limited impact on influenza vaccination, cervical cancer screening and mammography [140]. Small changes were attributed to low financial value, lack of physician awareness, competing incentives, and administrative burden [115]. Key quality indicators showed improvement in timely follow-up care, reduced time to depression improvement [148], increased quality care [119] and modest improvement in access to care for patients with chronic illness [92]. There was limited evidence on P4P incentives *targeted to the team*. Available evidence suggests that team incentives encourage collaboration and quality of care by improving the recording of patient diagnoses [91, 148].

### Team training

Team training interventions have a positive impact on team collaboration, provider satisfaction, and the development of high-functioning PC teams [94, 117]. These interventions emphasize the importance of aligning organizational culture and reward systems with training efforts.

### Resources

Adequate physical infrastructure [107, 112, 141] to support co-location [103, 111], financial resources for health human resources [151] and information technology [95] are essential for collaborative practice at the team level. Professional development opportunities contribute to team functioning by building trust and creating a sense of belonging [155].

### Protocols, guidelines, and agreements

Structured protocols, guidelines, and agreements at the team level are crucial for fostering effective team collaboration [143, 151] by clearly defining the roles, responsibilities, and tasks of team members [88, 96, 107, 110, 111, 150, 151].

### Team meetings

Team meetings promote effective team functioning [142], teamwork and collaboration [88, 102, 107, 110, 117]. Time devoted to meetings helps build relationships, clarify roles [88, 107, 151], enable communication [111], problem-solving [138], and build trust [87, 88, 94].

### Organizational culture

A positive organizational culture facilitates team collaboration and effectiveness [97, 103, 118, 145, 149]. Team interactions contribute to developing shared team vision, objectives and expectations for better patient outcomes [127]. Hierarchical cultures are barriers to collaboration [117] and teamwork, while flatter organizational structures empower individuals and promote shared decision-making [112].

Trust [135], respect [103, 118, 149], and effective leadership [112, 144, 155] are integral to fostering a culture of open communication, collaboration, innovation [155] and resilience [111].

### Intrinsic incentives and impact on outcomes

**Fig. 3:**
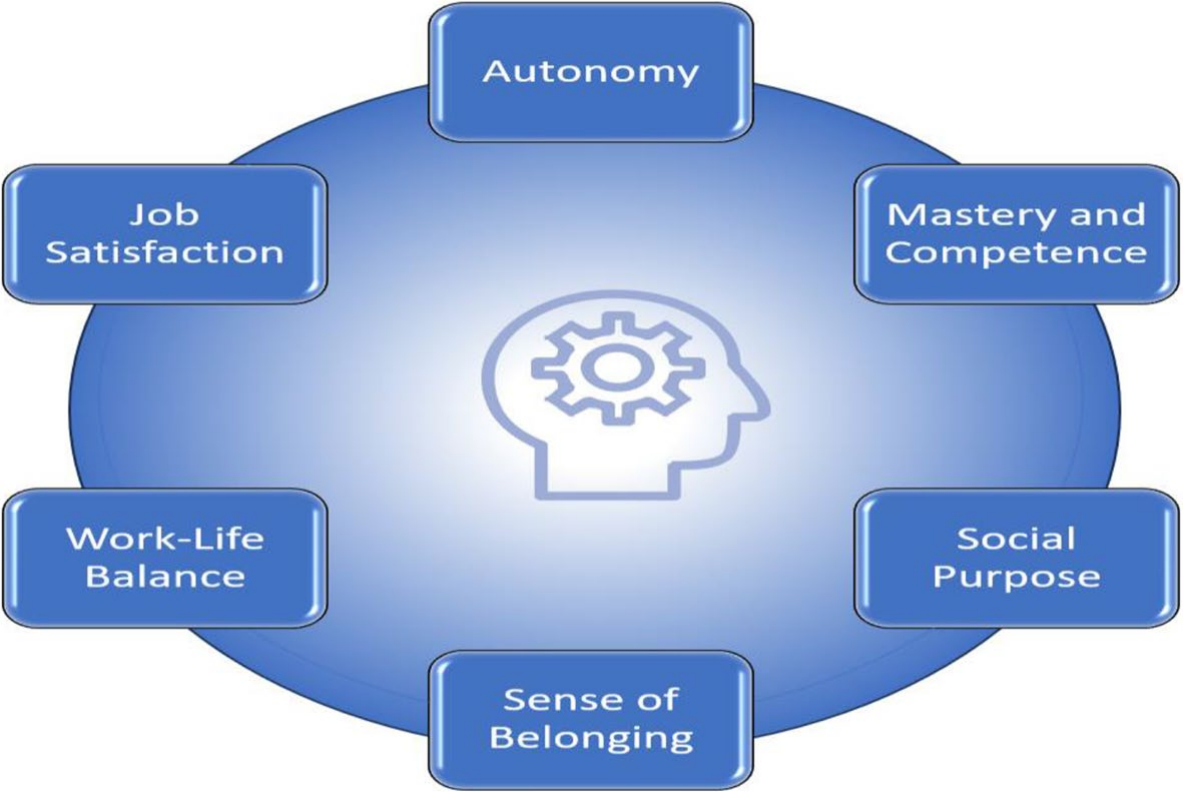
Intrinsic incentives and impact on outcomes

Twenty-two articles (*n* = 22/71,31%) identified intrinsic motivators (Fig. 3). Table 6 summarizes these articles in detail. The majority of these incentives were implemented at the individual level.

**Table 6:** Intrinsic in interprofessional primary care teams

Citation	Intrinsic Incentives (Level of Incentive, if specified or inferred)	Team Impact	Patient Impact	Provider Impact	System Impact
Burgess, J., Martin, A., & Senner, W. (2011). A framework to assess nurse practitioner role integration in primary health care. *Canadian Journal of Nursing Research Archive*, 22–41 [90]	Autonomy (Individual) Sense of Belonging (Individual)	Not Reported (NR)	Role recognition is perceived to result in collaboration with patients and the community. Role inclusion is perceived to improve the quality of care	Perceived to result in population management	Role alliance is perceived to result in more effective participation in practice innovations, health improvements and policy initiatives
Delva, D., Jamieson, M., & Lemieux, M. (2008). Team effectiveness in academic primary health care teams. *Journal of interprofessional care, 22*(6), 598–611 [96]	Sense of Belonging (Team)	Effectiveness	NR	NR	NR
Drew, P., Jones, B., & Norton, D. (2010). Team effectiveness in primary care networks in Alberta. *Healthcare quarterly* (Toronto, Ont.), 13(3), 33–38 [102]	Shared Purpose (Team)	Collaboration amongst the team	NR	NR	NR
Drummond, N., Abbott, K., Williamson, T., & Somji, B. (2012). Interprofessional primary care in academic family medicine clinics: implications for education and training. *Canadian Family Physician*, 58(8), e450-e458 [103]	Sense of belonging (trust and trusting relationship) (Team) Shared Purpose (Team)	Improved perception of team effectiveness is improved. Improved effective communication (team process)	NR	NR	NR
Hämel, K., & Vössing, C. (2017). The collaboration of general practitioners and nurses in primary care: a comparative analysis of concepts and practices in Slovenia and Spain. *Prim Health Care Res Dev*, 18(5), 492–506 [110]	Shared Purpose (Team)	Shared visions serve as a guiding force and motivation for collaborative efforts	Collaboration results in increased accessibility to a diverse range of expertise and skills among all team members, ultimately benefiting patient care	NR	NR
Harris, M. F., Advocat, J., Crabtree, B. F., Levesque, J. F., Miller, W. L., Gunn, J. M.,... Russell, G. M. (2016). Interprofessional teamwork innovations for primary health care practices and practitioners: evidence from a comparison of reform in three countries. *J Multidiscip Healthc*, 9, 35–46. 10.2147/JMDH.S97371 [111]	Job Satisfaction (Individual)	NR	NR	Improved personal performance	NR
Khazei, M., Shukor, A. R., & Biotech, M. (2020). A Novel Instrument for Integrated Measurement and Assessment of Intrinsic Motivation, Team Climate, and Burnout in Multidisciplinary Teams. *The Permanente Journal*, 24 [114]	Autonomy (Individual) Mastery (Individual) Purpose (Not specified) Wellbeing (Individual) Motivation (Individual)	A positive team climate contributes to better team performance. High intrinsic motivation leads to a more engaged, committed, and high-performing team. Low burnout levels help maintain high team performance	NR	High intrinsic motivation and low burnout levels contribute to higher job satisfaction, productivity, and retention	Staff retention of physicians
LaMothe, J., Hendricks, S., Halstead, J., Taylor, J., Lee, E., Pike, C., & Ofner, S. (2021). Developing interprofessional collaborative practice competencies in rural primary health care teams. *Nursing Outlook, 69*(3), 447–457 [117]	Alignment with Organizational Goals (Not specified)	Enabling interprofessional collaborative practice	NR	NR	NR
MacNaughton, K., Chreim, S., & Bourgeault, I. L. (2013). Role construction and boundaries in interprofessional primary health care teams: a qualitative study. *BMC health services research, 13*(1), 1–13 [121]	Job satisfaction (Individual) Role Clarity (Team) Alleviated Work Load (Individual) Autonomy (Individual)	Team members may experience job satisfaction when they can collaborate with colleagues, share their findings, and contribute to patient care in a meaningful way	NR	Increase professional satisfaction	NR
Markon, M.-P., Chiocchio, F., & Fleury, M.-J. (2017). Modelling the effect of perceived interdependence among mental HCPs on their work role performance. *Journal of interprofessional care, 31*(4), 520–528 [123]	Job Satisfaction (Individual) Autonomy (Individual)	Perceived work interdependence (task feature) positively relates to work role performance. Perceived collaboration (team process). Greater collaboration in decision-making (team process) due to knowledge sharing	As team functioning improves, so does the quality of patient care, leading to better patient outcomes	Improved skills, confidence in abilities, and job satisfaction due to increased collaboration and knowledge sharing	NR
Mohr, D. C., Young, G. J., Meterko, M., Stolzmann, K. L., & White, B. (2011). Job satisfaction of primary care team members and quality of care. *American Journal of Medical Quality, 26*(1), 18–25 [126]	Job Satisfaction (Individual)	Improved collaborative functioning	Job satisfaction positively associated with patient perception of care quality by using teams as the unit of analysis	NR	NR
Naccarella, L. (2009). General practitioner networks matter in primary health care team service provision. *Aust J Prim Health, 15*(4), 312–318 [129]	Competence (Individual)	Physicians work together in teams to solve problems and validate clinical decisions, legitimizing their actions when dealing with complex or chronic conditions	Improved decision-making processes can result in better patient care	NR	NR
Pereira, J. G., & Oliveira, M. A. d. C. (2018). Nurses’ autonomy in Primary Care: from collaborative practices to advanced practice. *Acta Paulista de Enfermagem, 31*, 627–635 [133]	Autonomy (Individual)	Fostering team collaboration	NR	NR	NR
Phipps-Taylor, M., & Shortell, S. M. (2016). More than money: motivating physician behavior change in accountable care organizations. The milbank quarterly, 94(4), 832–861 [66]	Autonomy (Individual) Sense of Belonging(Individual) Job Satisfaction(Individual)	Contributed to shared goals	NR	Embracing collaboration is difficult for physicians	NR
Pullon, S. (2008). Competence, respect and trust: Key features of successful interprofessional nurse-doctor relationships. *Journal of Interprofessional Care, 22*(2), 133–147. 10.1080/13561820701795069 [135]	Sense of Belonging (Individual)	Fostering functional relationships	NR	NR	NR
Pullon, S., Morgan, S., Macdonald, L., McKinlay, E., & Gray, B. (2016). Observation of interprofessional collaboration in primary care practice: a multiple case study. *Journal of interprofessional care, 30*(6), 787–794 [136]	Shared Purpose (Individual)	Fostering team collaboration	NR	NR	NR
Rioux-Dubois, A., & Perron, A. (2021). The integration of nurse practitioners into primary health care: Rethinking the negotiation of complex dynamics. *Recherche en soins infirmiers, 145*(2), 38–52 [137]	Autonomy (Individual)	Positive: Enhanced collaboration (team process). Negative: Perceived role confusion and anxiety, Perceived power asymmetries, Varying perceptions of roles and responsibilities	NR	Provider Impact: Positive: Higher job satisfaction, Sense of purpose, Professional growth. Negative: Perceived role confusion and anxiety, Perceived power asymmetries	NR
Rioux-Dubois, A., & Perron, A. (2022). Enacting primary healthcare interprofessional collaboration: a multisite ethnography of nurse practitioner integration in Ontario, Canada. *Journal of interprofessional care*, 1–9 [138]	Mastery (Individual) Sense of Belonging (Lack) (Individual) Job Satisfaction (Individual)	Power struggles are a barrier to collaboration	NR	Nurse practitioners (NPs) reported feeling having more purpose and job satisfaction due to their mastery and competence	
Shaw, A., De Lusignan, S., & Rowlands, G. (2005). Do primary care professionals work as a team: a qualitative study. *Journal of interprofessional care, 19*(4), 396–405 [144]	Shared Purpose (Team)	A lack of shared objectives and poor communication were all barriers to developing effective team working	NR	NR	NR
Shortell, S. M., Marsteller, J. A., Lin, M., Pearson, M. L., Wu, S.-Y., Mendel, P.,... Rosen, M. (2004). The Role of Perceived Team Effectiveness in Improving Chronic Illness Care. Medical Care, 42(11), 1040–1048 [145]	Shared Purpose (Individual)	Enabling interprofessional collaborative practice	NR	NR	NR
Song, H., Ryan, M., Tendulkar, S., Fisher, J., Martin, J., Peters, A. S.,... Singer, S. J. (2017). Team dynamics, clinical work satisfaction, and patient care coordination between primary care providers. *Health Care Management Review, 42*(1), 28–41 [146]	Sense of Belonging (Individual)	Improved team dynamics due to social inclusion, psychological safety, and productivity	Improved patient care coordination between teams resulting from better team dynamics	Greater clinical work satisfaction for attending clinicians relying on colleagues for consistent care. Resident physicians appreciate the positive aspects of team dynamics and increased work satisfaction	NR
Wilson, D. R., Moores, D. G., Lyons, S. C. W., Cave, A. J., & Donoff, M. G. (2005). Family physicians’ interest and involvement in interdisciplinary collaborative practice in Alberta, Canada. *Prim Health Care Res Dev, 6*(3), 224–231 [151]	Work-life balance (Individual)	Perceived that work**-**life quality could be improved by sharing responsibilities	NR	NR	NR

### Autonomy

Empowering individual PC providers with decision-making authority, control over work processes, and ownership of patient care tasks is essential for effective interprofessional collaboration, team interdependence, and high-performing PC teams [90, 114, 121, 123, 133, 137]. Power structures associated with designations (e.g., most responsible providers) hinder collaboration [138]. Delegating tasks by physicians to non-physician staff [66] and interchangeable roles can lead to power struggles within the team.

### Mastery and competence

Mastery and competence are powerful motivators at the individual level [66] that drive high-performing PC teams [114, 129, 137] and contribute to provider satisfaction [137]. Physicians find motivation in using performance data and monitoring progress over time to achieve mastery in their roles [66].

### Social purpose

A deep-seated sense of social purpose and commitment to positively impact patients and colleagues drive provider engagement [66], commitment to teams [102, 110, 114, 136], and dedication to patient needs. Purpose occurs at both the individual and team levels. When social purpose is described, it is often based on the team's vision and addressed by managers [110].

### Sense of belonging

Fostering a sense of belonging within teams plays a pivotal role in enhancing team dynamics, communication [96, 103] and developing high-performing teams since it mitigates provider burnout at the individual level, improves provider satisfaction and enhances patient care coordination [146]. PC providers emphasize the importance of feeling psychologically safe and being socially included within their teams. This sense of belonging is often perceived as more important to physicians than financial incentives [66].

### Work-life balance

Maintaining a healthy work-life balance at the individual level, achieved through workload sharing and role interchangeability [121] is crucial for quality of life, job satisfaction [151], and job performance [114]. Interchangeable roles reduce the individual workloads of team members, reducing burnout rates and increasing job satisfaction, consequently increasing productivity and improving provider retention [114].

### Job satisfaction

Collaborative teamwork significantly enhances job satisfaction at the individual level [155], which, in turn, has a profound impact on work performance and the quality of patient care [126].

## Discussion

Understanding the impact of payment remuneration models and extrinsic and intrinsic motivators on outcomes is crucial for optimizing PC service delivery. The structuring of PC models varies significantly across countries, particularly concerning remuneration systems, which in turn influence the implementation and efficacy of these models. This review found that non-FFS funding models, such as salaried models, are perceived to enhance team collaboration among healthcare providers, as evidenced by qualitative studies that highlight the effectiveness of non-hierarchical payment structures in fostering interdisciplinary cooperation. These findings have relevance to countries such as Canada, where FFS payment arrangements are the dominant model [156], which incentivize volume over value, often discouraging referrals to non-physician providers, undermining team collaboration and consequently influencing the spread of interprofessional teams [157]. Research indicates that FFS can create financial hierarchies that limit integrated care approaches [152, 158]. This trend in payment models is different from other countries, such as the United Kingdom [159], Netherlands [160] and Norway [161], which have implemented alternative payment models in team-based settings. A recent Milbank Quarterly paper by Aggarwal and colleagues noted that several contextual factors influence how models are implemented in different countries. These factors include federalism, policy legacies on professional practice and remuneration, embedded power structures and dynamics between professions, the degree of financial investments and the state of the evidence [4].

Policymakers, medical associations, and stakeholders should implement or expand, adapt and co-design alternative payment models, including salaried or blended salary/capitation models in team models. To prevent the unintended consequences of cream skimming in capitation models or lack of productivity in salaried models, these models should be accompanied by risk-adjustment formulas as well as clear and enforceable accountability processes for the organization, administration, and providers, which are linked to performance. Since many studies focus on process evaluations, investments must be made in rapid, robust, and timely outcomes evaluations of PC models to help inform decisions about improvement and expansion.

P4P incentives are the most common extrinsic incentives offered to physicians in team-based care models, and their impact is mixed. We found few studies that examined the impact of P4P incentives where the team is rewarded for performance based on various metrics. This approach recognizes the contributions of non-physician providers within teams, who often inherit the work associated with P4P incentives. Policymakers and medical associations should consider piloting P4P programs that focus on incentives for the team. The metrics and associated incentives should be developed in consultation with stakeholders representing the interprofessional team to build consensus and adoption. Case studies from existing P4P initiatives, such as the Medicare Shared Savings Program in the United States and the UK’s Quality and Outcomes Framework, indicate rewards and performance evaluation systems for teams should be based on valid, reliable, feasible and important performance measures which are easy to understand, allow team members to feel in control over their measured performance and objective and are inclusive of all members [159, 162]. These incentives should be carefully designed to avoid unintended consequences that lead to a focus on specific conditions or populations and based on objective and measurable data [163] and focus on metrics related to teamwork, patient satisfaction and system outcomes, such as reductions in emergency room visits, improved access to care, and cost-effectiveness. Investments in team training should accompany the program. Regular assessment of the program will allow it to remain aligned with evolving healthcare goals, promote team collaboration [116, 164], and permit decision-making on widespread implementation.

This review also demonstrates that PC teams must be adequately supported by extrinsic incentives to function optimally. In the US, healthcare organizations have integrated advanced information technology systems, enhancing communication, data sharing, and coordination among team members, ultimately leading to improved patient care [165]. Similarly, other countries, including Australia and the UK, have implemented strategies ensuring access to sufficient physical space for team meetings, thereby promoting effective teamwork [166]. Globally, teams have been resourced by many disciplines, including physicians, allied health professionals and support workers [1]. In the United States, advanced medical office assistants and panel managers can be part of the core team [1, 22, 167].

To foster collaboration, consensus-based protocols, policies and guidelines should be developed by professional colleges and associations representing different disciplines with jurisdictions that clearly articulate the roles, responsibilities and tasks of different PC providers.

Investments are also needed in team and leadership training, as exemplified by the UK’s leadership training for healthcare providers [168] and the US’s TeamSTEPPS program, which has been shown to improve team dynamics and communication, enhancing patient care [169]. Salas et al. highlight that team training programs, when well-designed and implemented, can significantly improve communication, coordination, and cohesion within healthcare teams, leading to better patient outcomes and increased job satisfaction among providers [170]. Furthermore, Weaver et al. found that team training is associated with improvements in both teamwork processes and clinical performance, reinforcing the need for structured training programs in healthcare settings [171].

Intrinsically motivated individuals tend to outperform extrinsically motivated individuals, even when their abilities are comparable [172]. Research indicates that when providers feel they have authority and control over their work processes, they exhibit higher levels of engagement and collaboration [173, 174]. Feeling valued and included in a team fosters collaboration [175] and less provider burnout [176]. Furthermore, performance improves when team members feel psychologically safe to voice concerns and share ideas without fear of negative consequences [177, 178]. Clinical and administrative leads have an important role in fostering a positive organizational culture in which there is a shared vision, clear articulation of roles, a sense of belonging, and mutual trust and respect between providers. Thus, it is crucial to recruit and adequately compensate high-quality and experienced professional leaders and managers with the knowledge and skills to build and support high-performing PC teams. In addition, a co-dyad approach will be important for leading a team [179].

Since providers are motivated by performance, policymakers should consider implementing a public reporting system for performance metrics, as done in New Zealand and the UK, to increase transparency, improve accountability, and motivate providers to enhance performance [180, 181]. Organizational behaviour literature also suggests team metrics should be part of regular reporting so teams are motivated to improve their collaboration, communication, and overall performance [182]. A performance measurement framework for PC teams should be co-designed and implemented to assess team functioning and enable routine reporting for PC team models. PC providers should be involved in the design and ongoing development of public reporting to enable buy-in for the initiative. External motivators (e.g., compensation) can complement and reinforce PC teams' internal motivation for teamwork (e.g., cohesion) [94].

### Limitations

Limitations of this study include the inclusion of English-only language documents, potentially excluding relevant studies and limiting the scope of the analysis. In addition, the restriction of the grey literature search to the first 100 pages of results may have resulted in the omission of additional relevant but less readily accessible sources beyond the 100-page cut-off. Consequently, some less widely disseminated grey literature may not have been captured, potentially impacting the comprehensiveness of findings. The categorization of intrinsic and extrinsic incentives was based on a literature review, which may have excluded specific extrinsic or intrinsic motivators. Moreover, the heterogeneity among included studies, stemming from differences in design, settings, and outcome measures, poses challenges in generalizing findings. Specifically, some studies employed intervention designs with multiple measurement periods and control conditions, while others were cross-sectional or non-interventional qualitative studies. This diversity in study design can impact the interpretation of findings. Future research should aim to build on the existing literature by employing robust study designs that can more effectively evaluate the long-term impacts of various remuneration models and incentive structures on primary care teams.

## Conclusion

This study underscores the need for a holistic approach, incorporating remuneration models and both extrinsic and intrinsic incentives, to maximize the potential of interprofessional PC teams. Policymakers, medical associations, and stakeholders should implement non-FFS provider payment models, such as blended or salaried payments, to foster collaboration and performance. This should be accompanied by the piloting of P4P incentives program for teams. Investments must also be made in capital that allows for sufficient physical space, health human resources, information technology and digital tools, and professional development opportunities. Interprofessional teams should have access to team training opportunities and highly effective dyad leadership models. To enable teamwork, stakeholders representing interprofessional teams must come together to develop consensus-based, clear and concise protocols and guidelines on the scope of practice, roles, and responsibilities to enhance team collaboration. Since providers are motivated by mastery, robust performance measurement systems with regular public reporting on co-designed performance outcomes can promote accountability and transparency. As the landscape evolves, continuous research and evaluation will be crucial to ensure the optimization of teamwork and healthcare delivery in PC settings. Future studies should explore the long-term impacts of these interventions on team, provider, patient and system outcomes.

## Supplementary Information

Supplementary Material 1. MMAT Assessment.

## Data Availability

Data is provided within the manuscript or supplementary information files.

## Abbreviations

CHC: Community Health Centers
CPAT: Collaborative Practice Assessment Tool
FFS: Fee-for-Service
FHTs: Family Health Teams
GPs: General Practitioners
MMAT: Mixed Methods Appraisal Tool
NR: Not Reported
PC: Primary Care
P4P: Pay-for-Performance
PRISMA-ScR: Preferred Reporting Items for Systematic Reviews and Meta-Analyses extension for scoping reviews
UK: United Kingdom
USA: United States of America
