# Supplementary material for: The impact of remuneration, extrinsic and intrinsic incentives on interprofessional primary care teams: results from a rapid scoping review
Source: BMC Prim Care. 2025 Feb 4;26:25. doi: 10.1186/s12875-024-02653-5 (PMC11796142; doi:10.1186/s12875-024-02653-5)
Supplement: Supplementary file 1 [file 12875_2024_2653_MOESM1_ESM.docx]

Ovid MEDLINE(R) ALL <1946 to November 23, 2022>

Date Last Searched: 07.12.22

[https://proxy.library.mcgill.ca/login?url=http://ovidsp.ovid.com/ovidweb.cgi?T=JS&NEWS=N&PAGE=main&SHAREDSEARCHID=2huydmbBYrp0oGR7dGCS4UyJL4EchenYQEOBZtTA6ynFXvi2fpXCwzTw8QepSxH0j](https://proxy.library.mcgill.ca/login?url=http://ovidsp.ovid.com/ovidweb.cgi?T=JS&NEWS=N&PAGE=main&SHAREDSEARCHID=2huydmbBYrp0oGR7dGCS4UyJL4EchenYQEOBZtTA6ynFXvi2fpXCwzTw8QepSxH0j) 

1	patient care team/ and (exp.interprofessional relations/ or cooperative behavior/ or primary 	care nursing/ or physicians, primary care/)	13,229

2	Primary health care/ and (exp.interprofessional relations/ or cooperative behavior/)	2868

3	Primary health care/ and (interprofessional or interdisciplinary or multidisciplinary).tw,kf.	2906

4	patient care team/ and (interprofessional or interdisciplinary or multidisciplinary).tw,kf.	18362

5	((interprofessional or interdisciplinary or multidisciplinary) adj3 (team* or network*)).tw,kf.	39775

6	((multidisciplinary or interdisciplinary or interprofessional) adj3 ('health team*' or 'care team*' 	or team*)).tw,kf.	39100

7	Nurse Practitioners/ and (interprofessional or interdisciplinary or multidisciplinary).tw,kf.	533

8	Family Practice/ and (interprofessional or interdisciplinary or multidisciplinary).tw,kf.	736

9            or /1-8   61882

10	Remuneration/ or Reimbursement Mechanisms/ or Fee-for-Service Plans/ or Capitation Fee/ or 	"Fees and Charges"/ or Fees, Medical/ or Reimbursement, Incentive/ or Models, Economic/ or 	Efficiency, Organizational/	70508

11	(blended adj2 capitation).tw,kf.	29

12	(team adj2 incentive*).tw,kf.	40

13	(pay adj2 performance).tw,kf.	2441

14	((financial or pay) adj2 bonus*).tw,kf.	73

15	(financial adj2 (compensation or model* or arrangement* or structure* or incentive*)).tw,kf.	7434

16	((extrinsic or intrinsic) adj2 (incentive* or motivation)).tw,kf.	2546

17	(psychological adj2 need*).tw,kf.	4039

18	Professional Autonomy/	9694

19	((paid or paternity or parental or maternity) adj2 leave).tw,kf.	1659

20	"Salaries and Fringe Benefits"/ or Job Satisfaction/ or Personal Satisfaction/	65537

21	'professional development'.tw,kf.	12867

22	(opportunit* adj3 advancement).tw,kf.	571

23	(recreational adj2 plan*).tw,kf.	43

24	(work adj2 satisfaction).tw,kf.	2232

25	or/10-24	170557

26	9 and 25	1879

27	limit 2 to yr=”2000 -Current”	1661
